# Mutation of Tyr137 of the universal *Escherichia coli* fimbrial adhesin FimH relaxes the tyrosine gate prior to mannose binding

**DOI:** 10.1107/S2052252516016675

**Published:** 2017-01-01

**Authors:** Said Rabbani, Eva-Maria Krammer, Goedele Roos, Adam Zalewski, Roland Preston, Sameh Eid, Pascal Zihlmann, Martine Prévost, Marc F. Lensink, Andrew Thompson, Beat Ernst, Julie Bouckaert

**Affiliations:** a Institute of Molecular Pharmacy, Pharmacenter, University of Basel, Klingelbergstrasse 50-70, CH-4056 Basel, Switzerland; b University of Lille, CNRS UMR8576 UGSF (Unité de Glycobiologie Structurale et Fonctionnelle), 59000 Lille, France; c Structure et Fonction des Membranes Biologiques, Université Libre de Bruxelles (ULB), Brussels, Belgium; d Synchrotron SOLEIL, l’Orme de Merisiers, Saint-Aubin BP48, Gif-sur-Yvette CEDEX, France

**Keywords:** *Escherichia coli* infection, FimH adhesin, tyrosine gate, mutations, crystals, thermodynamics, peptide torsions, heptyl mannose, biphenyl mannose, molecular dynamics, protein structure, molecular recognition, X-ray crystallography

## Abstract

The most prevalent diseases manifested by *Escherichia coli* are acute and recurrent bladder infections and chronic inflammatory bowel diseases such as Crohn’s disease. *E. coli* clinical isolates express the FimH adhesin, which consists of a mannose-specific lectin domain connected *via* a pilin domain to the tip of type 1 pili. Although the isolated FimH lectin domain has affinities in the nanomolar range for all high-mannosidic glycans, differentiation between these glycans is based on their capacity to form predominantly hydrophobic interactions within the tyrosine gate at the entrance to the binding pocket. In this study, novel crystal structures of tyrosine-gate mutants of FimH, ligand-free or in complex with heptyl α-d-*O*-mannopyranoside or 4-biphenyl α-d-*O-*mannopyranoside, are combined with quantum-mechanical calculations and molecular-dynamics simulations. In the Y48A FimH crystal structure, a large increase in the dynamics of the alkyl chain of heptyl α-d-*O*-mannopyranoside attempts to compensate for the absence of the aromatic ring; however, the highly energetic and stringent mannose-binding pocket of wild-type FimH is largely maintained. The Y137A mutation, on the other hand, is the most detrimental to FimH affinity and specificity: (i) in the absence of ligand the FimH C-terminal residue Thr158 intrudes into the mannose-binding pocket and (ii) ethylenediaminetetraacetic acid interacts strongly with Glu50, Thr53 and Asn136, in spite of multiple dialysis and purification steps. Upon mutation, pre-ligand-binding relaxation of the backbone dihedral angles at position 137 in the tyrosine gate and their coupling to Tyr48 *via* the interiorly located Ile52 form the basis of the loss of affinity of the FimH adhesin in the Y137A mutant.

## Abstract 2

The role of the two tryrosine gating residues in the binding affinity of FimH for substituted mannosides is investigated using a combination of mutagenesis, X-ray crystallography, affinity measurements and molecular simulation. In contrast to the Y48A mutation, it is clearly shown that the Y137A mutation relaxes the binding site prematurely, whereby the stringent selectivity of the FimH lectin for mannose is disrupted and the binding affinity decreases.

## 1. Introduction   

*Escherichia coli* infections are the most common infections in the human urinary tract (Goneau *et al.*, 2015 ▸; Hooton & Stamm, 1997 ▸), but *E. coli* is also involved in many other infections, for example Crohn’s disease-associated microbial dysbiosis (Darfeuille-Michaud *et al.*, 2004 ▸). These Gram-negative bacteria express type 1 fimbriae on their surface: long, hair-like proteinaceous organelles assembled at the outer membrane usher with the assistance of the periplasmic chaperone FimC (Choudhury *et al.*, 1999 ▸). The FimH adhesin is located at the tip of the fimbriae and consists of an N-terminal lectin domain and a C-terminal pilin domain that connects FimH to the rest of the pilus *via* FimG followed by FimF (Le Trong, Aprikian, Kidd, Thomas *et al.*, 2010 ▸). The FimH lectin binds specifically to mannose moieties displayed on glycoproteins at the luminal surface of epithelial cells (Gouin *et al.*, 2014 ▸). Attachment of bacteria to the bladder epithelium is followed by their internalization and subsequent biofilm formation inside the urothelial cells (Anderson *et al.*, 2004 ▸), which facilitates their persistence within the urinary tract. FimH-mediated binding is enhanced under shear force (Thomas *et al.*, 2002 ▸). The origin of this binding enhancement is the disruption of the quaternary interactions between the lectin and pilin domains of FimH by shear force, morphing the lectin into an elongated β-sandwich with high affinity (Le Trong, Aprikian, Kidd, Forero-Shelton *et al.*, 2010 ▸). When the shear force is alleviated, the number of interactions between the lectin and pilin domains increases again at the expense of FimH affinity towards the ligand (Aprikian *et al.*, 2007 ▸).

Antibiotics are the standard treatment for *E. coli* infections; however, their long-term use promotes the development of microbial resistance, leading to recurrent infections and thus accounting for significant morbidity (Geerlings *et al.*, 2014 ▸). Anti-adhesive drugs prevent the binding and ensure the clearance of type 1 piliated *E. coli* from epithelial linings (Sivignon *et al.*, 2015 ▸). They present a promising alternative treatment to antibiotics, in particular because anti-adhesives are unlikely to induce bacterial resistance as they do not interfere with the bacterial metabolism: their effect is neither bacteriostatic nor bacteriolytic. The knowledge needed for the design of FimH antagonists is traditionally based on crystal structures (Choudhury *et al.*, 1999 ▸; Bouckaert *et al.*, 2013 ▸; Brument *et al.*, 2013 ▸; Cusumano *et al.*, 2011 ▸; Firon *et al.*, 1987 ▸; Hartmann & Lindhorst, 2011 ▸; Klein *et al.*, 2010 ▸; Han *et al.*, 2012 ▸). The earliest report on potential anti-adhesives described the FimC chaperone holding the FimH adhesin that accommodated cyclohexylbutanoyl-*N*-hydroxyethyl-d-gluc­amide (C-HEGA) at the tip. The linear glucosamide of C-HEGA, which was used as an additive in the crystallization condition, gave the first indications of the position of the mannose-binding site and its interactions (Choudhury *et al.*, 1999 ▸). By crystallizing the FimH–FimC complex in the presence of methyl α-d-mannopyranoside, the first FimH structures in complex with its most specific sugar, mannose, appeared shortly afterwards (Hung *et al.*, 2002 ▸). Mannose binds in a deep and polar pocket at the N-terminal end of FimH, in which the amino acids Phe1, Asn46, Asp47, Asp54, Gln133, Asn135, Asp140 and Phe142 create a dense network of 12 hydrogen bonds with each hydroxyl group of the pyran­ose ring except that at the α-anomeric position of mannose. More recent crystal structures of the FimH lectin are complexed with O-linked (Fiege *et al.*, 2015 ▸), N-linked (Brument *et al.*, 2013 ▸; Chalopin *et al.*, 2016 ▸) and C-linked (de Ruyck *et al.*, 2016 ▸) mannosides that are less sensible to glycosidases and better suited for therapeutic use (Mydock-McGrane *et al.*, 2016 ▸).

In the first high-resolution structures of the FimH lectin domain, *n*-butyl α-d-mannopyranoside was serendipitously found strongly bound in the mannose-binding site (Bouckaert *et al.*, 2005 ▸). These structures showed that the butyl aglycon extending from the mannose ring interacts with Ile52, which bridges residues Tyr48 and Tyr137. These tyrosine residues form the so-called tyrosine gate. The tyrosine gate has recently attracted great interest because of its potential to generate nanomolar affinities for mannosides conjugated to hydrophobic aglycons through the formation of favourable van der Waals and stacking interactions within the gate. Aglycon substituents either travel through the gate like natural high-mannose N-linked glycans (Wellens *et al.*, 2008 ▸), for example the biphenyl aglycon moiety of a C-linked mannoside (de Ruyck *et al.*, 2016 ▸), or interact in multiple possible stacking modes with Tyr48 alone (Han *et al.*, 2010 ▸) or with both tyrosines of the gate (Wellens *et al.*, 2012 ▸; Kleeb *et al.*, 2015 ▸).

To establish a detailed interaction profile of the tyrosine-gate residues and to rationalize the binding affinities of various FimH antagonists, we generated single-residue FimH mutants in which one of the two tyrosine-gate residues was mutated to alanine (Y48A and Y137A). The effect of these mutations on the binding of three synthetic ligands was tested by protein crystallography, by measuring affinities and by molecular-simulation studies. These data highlight the importance of Tyr137, as its mutation clearly alters the binding properties of the FimH lectin. The ligand-free state of Y137A FimH permits the C-terminal carboxylate of a neighbouring molecule to enter its mannose-binding pocket. This mutant also binds a buffer molecule in a shallow surface pocket near Glu50, Thr53 and Asn136. In the ligand-free state of Y137A FimH, several of the binding-site residues (including Asn136, Tyr137 and Tyr48) already sample backbone dihedral angles that are normally only found in wild-type FimH upon binding of the mannose. Simulations indicate a dynamic coupling of Tyr137 with Tyr48 *via* Ile52. Altogether, these data can explain the significantly lower binding affinity towards the compounds tested here in the Y137A mutant compared with the wild-type lectin. The results presented here thus considerably improve our understanding of FimH–mannoside interaction and guide a better design strategy for FimH antagonists.

## 2. Materials and methods   

### 2.1. Reagents   

Bacto Yeast Extract, Bacto Agar and Bacto Tryptone were purchased from Becton Dickinson (Basel, Switzerland) and used for the preparation of LB (Luria–Bertani) culture medium. Isopropyl β-d-1-thiogalactopyranoside (IPTG) was obtained from Applichem (Darmstadt, Germany). Polymyxin B sulfate, HEPES [4-(2-hydroxyethyl)-piperazine-1-ethanesulfonic acid], oxalic acid, MgCl_2_, CaCl_2_, NaH_2_PO_4_ and imidazole were from Fluka (Buchs, Switzerland). Ampicillin, bovine serum albumin (BSA), ethylenediaminetetraacetic acid (EDTA), amino acids and BME vitamin mix were obtained from Sigma. The sugars 1,5-anhydro-d-mannitol (AM), *n*-heptyl α-d-mannopyranoside (HM) and the 4-biphenyl α-d-mannopyranoside (BF) were synthesized as described previously (Pang *et al.*, 2012 ▸).

### 2.2. Cloning of tyrosine-gate mutants   

The FimH lectin-domain construct linked to the thrombin cleavage site (Th) and a His_6_ tag (6His) was generated as described by Rabbani *et al.* (2010 ▸). The Y48A and Y137A mutants were generated by the overlap extension PCR method using the plasmid encoding the wild type (WT) as a template. The inserts were digested with HindIII and XbaI restriction enzymes, gel-purified and subsequently ligated into the corresponding cloning site of pDsbA3 expression vector. The vectors were then transformed into *E. coli* DH5α chemo-competent cells (Novagen, Lucerne, Switzerland). After plasmid isolation and restriction control, the correctness of the constructs was confirmed by DNA sequencing (Microsynth, Balgach, Switzerland). Finally, the vectors were transformed into the protease-deficient *E. coli* strain HM 125 (Meerman & Georgiou, 1994 ▸) for protein expression.

### 2.3. Protein expression and purification   

Bacterial clones were grown at 30°C with vigorous shaking (300 rev min^−1^) in M9 minimal medium (Sambrook, 1989 ▸) supplemented with 2 m*M* MgSO_4_, 0.1 m*M* CaCl_2_, 20% glucose, 10 µ*M* of each amino acid, BME vitamin mix (Sigma, Buchs, Switzerland) and 100 µg ml^−1^ ampicillin. When an OD_600_ of 0.8 was reached, the cells were induced with 1 m*M* IPTG and cultivated for a further 16 h at 30°C and 300 rev min^−1^. The cells were then cooled on ice for 5 min and harvested by centrifugation at 5000 rev min^−1^ for 20 min at 4°C. The pellet was suspended in a cold solution of 50 m*M* Tris–HCl pH 7.5, 150 m*M* NaCl, 5 m*M* EDTA, 1 mg ml^−1^ polymyxin B sulfate (lysis buffer) and stirred for 2 h at 4°C. After centrifugation at 11 000 rev min^−1^ for 20 min at 4°C, the supernatant (periplasmic extract) was dialyzed overnight against 50 m*M* NaH_2_PO_4_, 300 m*M* NaCl, 10 m*M* imidazole pH 8 (binding buffer) and applied onto an Ni–NTA column (Sigma, Buchs, Switzerland) attached to a BioLogic fast protein liquid chromatography system (Bio-Rad, Reinach BL, Switzerland). The column was washed with binding buffer and was subsequently eluted with 50 m*M* NaH_2_PO_4_, 300 m*M* NaCl, 250 m*M* imidazole pH 8 (elution buffer). The fractions containing FimH were pooled and dialyzed against 20 m*M* HEPES, 150 m*M* NaCl, 1 m*M* CaCl_2_ pH 7.4 (assay buffer). The purity of the protein was verified by SDS–PAGE analysis and the amount was determined by HPLC (Bitsch *et al.*, 2003 ▸) using BSA as a standard. Aliquots of the proteins could be stored for up to three months at 4°C. For long-term storage, the proteins were frozen at −80°C without additives. For protein crystallization purposes, a FimH lectin construct (amino acids 1–158) without any tag was generated and was purified by ion-exchange chromatography (Vetsch *et al.*, 2002 ▸).

### 2.4. Circular-dichroism analysis   

The far-UV region of a protein circular-dichroism (CD) spectrum delivers spectral features that are directly relevant to the secondary structure of the protein. To analyze the effect of the mutations on the secondary structure of FimH, CD spectra of the wild type and mutants were recorded. The proteins were diluted to a concentration of 10 µ*M* in 10 m*M* sodium phosphate buffer pH 7.4 and CD spectra were measured at 25°C using a thermostat-controlled 0.1 cm cell. The spectra were recorded between 190 and 250 nm with 2 nm bandwidth and ten scans per sample on a Jasco J-720 spectrophotometer with a PFD-350S temperature controller connected to the J-700 software program. CD spectra were corrected for background by subtracting buffer spectra and the CD signal was converted to residue ellipticity ([θ]_MRW_ in deg cm^2^ dmol^−1^).

### 2.5. Fluorescence measurements   

The conformational stability was verified by monitoring the guadinium chloride (GdmCl)-induced unfolding profiles of the wild type and mutants. Prior to spectroscopic measurements, the samples (10 µ*M*) were incubated with GdmCl concentrations ranging from 0 to 6 *M* for 24 h at 25°C. The effective GdmCl concentrations were determined from the refractive indices of the samples (Pace, 1986 ▸). Fluorescence measurements were performed on a Hitachi F4500 fluorescence spectrometer at 25°C using an excitation slit of 2.5 nm and an emission slit of 5 nm. All samples were excited at λ = 280 nm and the spectra were recorded at a scan speed of 10 nm min^−1^. The fluorescence emission for equilibrium unfolding experiments was followed at λ = 350 nm (tryptophan fluorescence). Fluorescence spectra were corrected for background by subtracting buffer spectra. All transitions were evaluated according to a two-state model using the linear extrapolation method and normalized.

### 2.6. Protein mass-spectrometric analysis   

The exact molecular weights of wild-type FimH lectin and the tyrosine mutants were determined by electrospray ionization mass spectrometry.

### 2.7. Competitive binding assay   

To evaluate the affinity of the mutants, a previously described competitive binding assay (Rabbani *et al.*, 2010 ▸) was used. Briefly, 100 µl per well of a 10 µg ml^−1^ solution of FimH in 20 m*M* HEPES, 150 m*M* NaCl, 1 m*M* CaCl_2_ pH 7.4 (assay buffer) was coated onto a MaxiSorp Nunc (Roskilde, Denmark) 96-well microtitre plate overnight at 4°C. The coating solution was discarded and the wells were blocked with 150 µl per well of 3% bovine serum albumin in assay buffer for 2 h at 4°C. After three washing steps with assay buffer (150 µl per well), a serial dilution of the test compound (50 µl per well) in assay buffer consisting of 5% dimethyl sulfoxide and Manα1,3(Manα1,6)Manβ1,4GlcNAcβ1,4GlcNAcβ-PAA-biotin glycopolymer (Lectinity, Moscow, Russia) saturated with peroxidase-coupled strept­avidin (50 µl per well of a 0.5 µg ml^−1^ solution) was added. The plates were incubated for 3 h at 25°C and 350 rev min^−1^ and then carefully washed four times with 150 µl per well assay buffer. Colour was developed using 100 µl 2,2′-azino-bis(3-ethylbenzothiazoline-6-sulfonic acid) as the substrate per well. The reaction was stopped after 4 min by the addition of 2% aqueous oxalic acid before the optical density was measured at 415 nm on a microplate reader. The IC_50_ values of the compounds tested in duplicate were calculated with *GraphPad Prism* (GraphPad Software, La Jolla, USA). The IC_50_ defines the molar concentration of the test compound that reduces the maximal specific binding of glycopolymer to FimH lectin by 50%. The relative IC_50_ (rIC_50_) is the ratio of the IC_50_ of the test compound to the IC_50_ of *n*-heptyl α-d-mannopyranoside and was set to 1 for wild-type FimH.

### 2.8. Isothermal titration calorimetry   

A thermodynamic characterization of the interactions between WT, Y48A and Y137A FimH lectin and mannosides was obtained by isothermal titration calorimetry (ITC). The protein was dialyzed overnight at 4°C against assay buffer using Slide-A-Lyzer dialysis cassettes with 10 kDa cutoff (Thermo Fisher Scientific, Waltham, Massachusetts, USA). FimH concentrations of 10–27 µ*M* were used in the measurement cell. The ligands AM, HM and BF were diluted to a tenfold to 20-fold molar excess of the protein. The dilution enthalpy was determined by ligand in assay buffer titration. All measurements were performed with a MicroCal VP-ITC instrument (GE Healthcare, Northampton, Massachusetts, USA; sample-cell volume of 1.4523 ml) at 25°C, 307 rev min^−1^ stirring speed and 10 µcal s^−1^ reference power. The ligand was injected in 4–8 µl steps (38–66 injections in total) with a spacing of 10 min to ensure non-overlapping peaks. Sigmoidal binding curves with complete saturation were obtained at the end of each experiment. Baseline correction and peak integration were performed using *Origin* 7 (Origin­Lab, Northampton, Massachusetts, USA). Baseline subtraction and curve-fitting with the three variables *n* (molar ratio), *K*_d_ (dissociation constant) and Δ*H* (change in enthalpy) were performed using a global analysis with *SEDPHAT* v.10.40 (National Institutes of Health; Houtman *et al.*, 2007 ▸). The parameters Δ*G* (change in free energy) and Δ*S* (change in entropy) were calculated by introducing the measured *n*, Δ*H* and *K*_a_ into the formula Δ*G* = Δ*H* − *T*Δ*S* = −*RT*ln*K*_a_ = *RT*ln*K*_d_, where *T* is the absolute temperature (295.15 K for the measurements) and *R* is the universal gas constant (8.314 J mol^−1^ K^−1^). The 95% confidence intervals of the measurements were calculated for the two variables *K*_d_ and Δ*H*° with the one-dimensional error surface projection within *SEDPHAT*. The quantity *c* = Mt(0)*K*_d_^−1^, where Mt(0) is the initial macromolecule concentration, ranged between 10 and 600 for all experiments, thus being in a reliable measurement range (Wiseman *et al.*, 1989 ▸).

### 2.9. Crystallization, data collection and refinement   

Crystallization of the FimH lectin was accomplished by the sitting-drop vapour-diffusion method using Crystal Screen and Crystal Screen 2 from Hampton Research and The PGA Screen, PACT, MIDAS and 3D Structure Screen from Molecular Dimensions. Co-crystallization conditions of each FimH structure are given in Table 1 ▸, as well as data-processing and refinement statistics. Data were collected on PROXIMA 1 at SOLEIL, Saint-Aubin, France and PX14.2 at BESSY, Berlin, Germany. The structure determination was performed using molecular replacement in *Phaser* with ligand-free FimH (PDB entry 4auu; Wellens *et al.*, 2012 ▸) as a model. The ligands were parameterized using the *PRODRG* server (Schüttelkopf & van Aalten, 2004 ▸) and the crystal structures were refined using *BUSTER* (for low resolution and solvent mask; Smart *et al.*, 2012 ▸) and *PHENIX* (for high resolution and water picking; Adams *et al.*, 2002 ▸). Geometric evaluation and φ/ψ backbone torsion-angle readout were performed using *MolProbity* v.4.3 (Chen *et al.*, 2010 ▸).

### 2.10. Quantum-mechanical calculations   

The Asn135–Ser139 pentapeptide from wild-type FimH and from the Y137A mutant in complex with HM, as well as the Asn46–Glu50 pentapeptide from wild-type FimH and from the Y48A mutant in complex with HM, were extracted from PDB entries 4buq (WT), 5fs5 (Y137A) and 4ca4 (Y48A), respectively. Hydrogen atoms were placed and their positions were optimized at the B3LYP/6-31+G(d,p) level (for the wild type) or with 5fs5 (for Y137A and Y48A). Rigid potential energy surface scans (ten steps of 2.0° in both positive and negative directions) were performed for the rotation around the φ and ψ dihedral angles of Asn136, Tyr137 (Ala137) and Asn138 and also Asp47, Tyr48 (Ala48) and Pro49 at the M06-2X/6-311+G(d,p) level. All quantum-mechanical (QM) calculations were performed using *Gaussian* 09 (Frisch *et al.*, 2009 ▸).

### 2.11. Molecular dynamics simulations   

All molecular dynamics (MD) trajectories were generated in the isothermal–isobaric ensemble at 300 K with *NAMD*2.9 (Phillips *et al.*, 2005 ▸) using the CHARMM36 force field (MacKerell *et al.*, 1998 ▸, 2004 ▸; Vanommeslaeghe *et al.*, 2010 ▸; Guvench *et al.*, 2011 ▸; Mallajosyula & MacKerell, 2011 ▸). Long-range electrostatic interactions were calculated using the particle mesh Ewald method (Darden *et al.*, 1993 ▸). A smoothing function was applied to truncate short-range electro­static interactions. The Verlet-I/r-RESPA multiple time-step propagator (Tuckerman *et al.*, 1992 ▸) was used to integrate the equations of motion using time steps of 2 and 4 fs for short-range and long-range forces, respectively. All bonds involving H atoms were constrained using the *Rattle* algorithm (Andersen, 1983 ▸). Missing force-field parameters for BF and HM were generated with *CGenFF* (Vanommeslaeghe *et al.*, 2010 ▸). A single 50 ns MD simulation of BF and HM alone in water was subsequently performed.

The coordinates of the FimH lectin-domain crystal structure in either its ligand-free state (PDB entry 4auu; Wellens *et al.*, 2012 ▸), bound to HM (Table 1 ▸; PDB entry 4buq) or bound to BF (Table 1 ▸; PDB entry 5fwr) were used as initial coordinates. The Y48A or Y137A mutation was introduced *in silico* using the *mutate* tool of *VMD* (Humphrey *et al.*, 1996 ▸). Each of the nine systems was solvated and the ionic concentration was set to 0.15 *M* NaCl. All ionizable groups were assigned their standard protonation state as predicted by *PROPKA* (Bas *et al.*, 2008 ▸). Each molecular system comprised of a total of about 45 000 atoms. The equilibration was performed in three steps: (i) a 2.5 ns equilibration of the solvent (water and ions), (ii) a 2.5 ns equilibration in which only the protein backbone was fixed and (iii) an unrestrained 2.5 ns simulation. This was followed by three independent 50 ns MD production trajectories for each system. In addition, following the same protocol, two 50 ns production trajectories were generated using the crystal structures of Y48A and Y137A FimH with HM bound (PDB entries 4ca4 and 5fs5, Table 1 ▸) and of the Y137A mutant in its ligand-free state (PDB entry 5fx3, Table 1 ▸) to investigate whether the *in silico* generation of the mutations had an impact on the simulations.

The backbone dihedral angle values of residues 47–49 and 136–138 were extracted with *VMD* (Humphrey *et al.*, 1996 ▸) every 2 ps from all trajectories, allowing sufficient data for the calculation of a dihedral angle distribution. The most abundant conformations of the tyrosine gate were determined by clustering the trajectories of ligand-free FimH (PDB entry 4auu) using the *G_CLUSTER* tool from the MD suite *GROMACS* (Pronk *et al.*, 2013 ▸), based on the root-mean-square deviation (r.m.s.d.) matrix of the two tyrosine-gate residues Tyr48 and Tyr137. A total of 15 000 frames was used for the clustering, extracted every 10 ps using the *GROMOS* clustering algorithm (Daura *et al.*, 1999 ▸) with a cutoff of 1.5 Å. The representative FimH configuration of the cluster with the highest probability was used to screen for protein-residue interactions using *NCIPLOT* (Contreras-García *et al.*, 2011 ▸). The distance between Ile52 and residues Tyr48 and Tyr137, respectively, was measured as the distance between the two centres of mass of all side-chain heavy atoms of each residue. The flexibility of the HM and BF bound to FimH was assessed by computing the root-mean-square fluctuation (r.m.s.f.) difference (Δr.m.s.f.) of the ligands in the protein compared with its flexibility in water. The r.m.s.f. is a quantity describing the movement of each considered atom around the average structure and is defined as
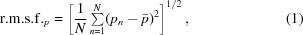
where *p_n_* is the position of an atom of interest in the frame *n*, 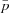 is the position of the same atom in the average structure and *N* is the total number of frames of the considered MD trajectory. As the mannose moiety of both ligands remains in a similar position during the trajectories, the r.m.s.f. was computed after alignment of the mannose ring using *VMD*. The backbone r.m.s.d. of the protein was computed using *VMD* and the secondary-structure content was computed with *STRIDE* (Heinig & Frishman, 2004 ▸) and averaged over the trajectories. Figures were prepared using *VMD*.

## 3. Results and discussion   

### 3.1. The tyrosine-gate mutant Y137A folds more readily towards a lower energy minimum   

In the last ten years, several FimH antagonists have been rationally designed, synthesized and evaluated for the treatment of *E. coli*-borne infections (Gouin *et al.*, 2014 ▸; Mydock-McGrane *et al.*, 2016 ▸; Klein *et al.*, 2010 ▸). These antagonists consist of a mannose monosaccharide α-glycosidically linked to various, predominantly aromatic, aglycones. The interaction of the aglycones with the tyrosine gate formed by two tyrosines (Tyr48 and Tyr137) at the entrance to the FimH binding site contributes significantly to their affinity, for example with a 400-fold increase for HM compared with α-d-manno­pyranoside (Wellens *et al.*, 2012 ▸). To experimentally evaluate the individual contributions of these two aromatic residues, the corresponding alanine mutants (Y48A and Y137A) were generated here.

**Figure 1:**
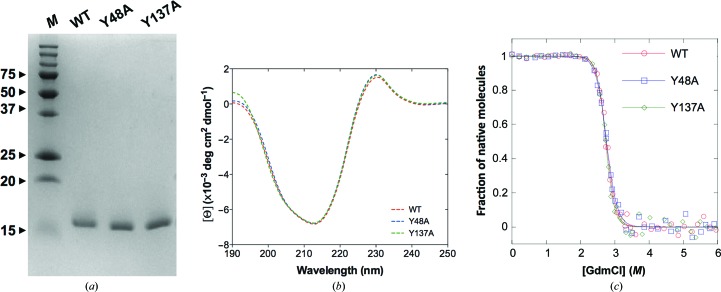
Characterization of wild-type FimH lectin and mutants. (*a*) Affinity-purified wild-type FimH and the tyrosine-gate mutants Y48A and Y137A were subjected to SDS–PAGE under reducing conditions on a 16% acrylamide/bisacrylamide SDS–PAGE gel. Lane *M*, molecular-weight marker (labelled in kDa). (*b*) CD spectra of WT FimH lectin and the Y48A and Y137A mutants. All samples were measured at 10 µ*M* concentration in 10 m*M* sodium phosphate buffer pH 7.4 and at 25°C using a thermostat-controlled 0.1 cm cell as described in §[Sec sec2]2. (*c*) GdmCl-dependent equilibrium unfolding profiles at 25°C and pH 7.4 were monitored by changes in fluorescence at 350 nm upon excitation at 280 nm. The transition midpoint values *D*_1/2_ are 2.75 *M* (WT), 2.77 *M* (Y48A) and 2.73 *M* (Y137A) GdmCl.

Wild-type FimH and the two mutants were expressed in the protease-deficient *E. coli* HM 125, extracted from the periplasm and purified to homogeneity (Fig. 1 ▸ *a*). Their exact molecular weights were confirmed by ESI-MS (Supplementary Fig. S1). Identical CD spectrum profiles (Fig. 1 ▸ *b*) indicate that the mutants in the native conformation retain a similar secondary structure to the wild type (WT). Furthermore, the stability of the mutants is preserved, as verified by GdmCl-unfolding experiments. The conformational transition curves of the WT and the mutants show superimposable profiles, with similar transition midpoint values (Fig. 1 ▸ *c*). The free energy of folding and the folding cooperativity for the WT and the Y48A and Y137A mutants indicate that the Y48A mutant is the least stable protein, with the lowest folding cooperativity, whereas FimH may even be stabilized by the Y137A mutation (Table 2 ▸).

### 3.2. The tyrosine-gate FimH mutant Y137A displays a lower affinity for conjugated mannosides   

**Figure 2:**
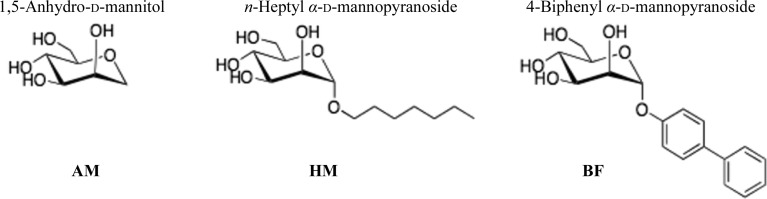
Chemical structures of AM, HM and BF.

The relative affinities of WT FimH lectin and the Y48A and Y137A mutants for AM, HM and BF (Fig. 2 ▸), determined by a competitive binding assay (Rabbani *et al.*, 2010 ▸), are reported as relative inhibition constants (rIC_50_), with the mannoside AM as the reference compound (Table 3 ▸). Moreover, the thermodynamic parameters for the binding of AM, HM and BF to WT FimH and the Y48A and Y137A mutants have been determined by ITC (Table 3 ▸).

In wild-type FimH, the mannosides exhibit largely enthalpy-driven binding, in agreement with previously reported results for other monomannoside and oligomannoside derivatives (Durka *et al.*, 2011 ▸; Wellens *et al.*, 2012 ▸) and for a series of biphenyl α-d-mannosides (Fiege *et al.*, 2015 ▸; Pang *et al.*, 2012 ▸). The affinity for AM is only slightly reduced in the mutants compared with the WT, although a decrease in the enthalpic contribution in Y48A is indicative of a loss of interactions. Because of the lack of an aglycon on AM, this reduced enthalpy cannot be understood in terms of a loss of direct contacts of the ligand with one or both tyrosines. In Y137A FimH, the enthalpic contribution to binding is further diminished, which is compensated for by a lower entropic cost, which is called enthalpy–entropy compensation. This also shows that although mutation of the tyrosine gate may negatively influence the binding of AM, it remains indifferent as to which tyrosine is mutated. The Y48A mutation in FimH has a similar effect on the affinity for HM and BF of FimH, *i.e.* the affinity values remain close, within a factor of 2.3–2.6, to those obtained for wild-type FimH (Table 3 ▸). This occurs again through an enthalpy–entropy compensation effect: the lack of potential to stack with the Tyr48 aromatic side chain is resolved by an increased entropic contribution. In stark contrast, the Y137A mutation results in a dramatic decrease in affinity towards HM and BF, by a factor of 7.1 and 5.1, respectively (Table 3 ▸). Here, the impact on the enthalpic change cannot be completely compensated for by a positive entropic change.

### 3.3. The crystal structure of ligand-free Y137A reveals an intrusion into the mannose-binding site and exposes an unexpected binding site nearby   

**Figure 3:**
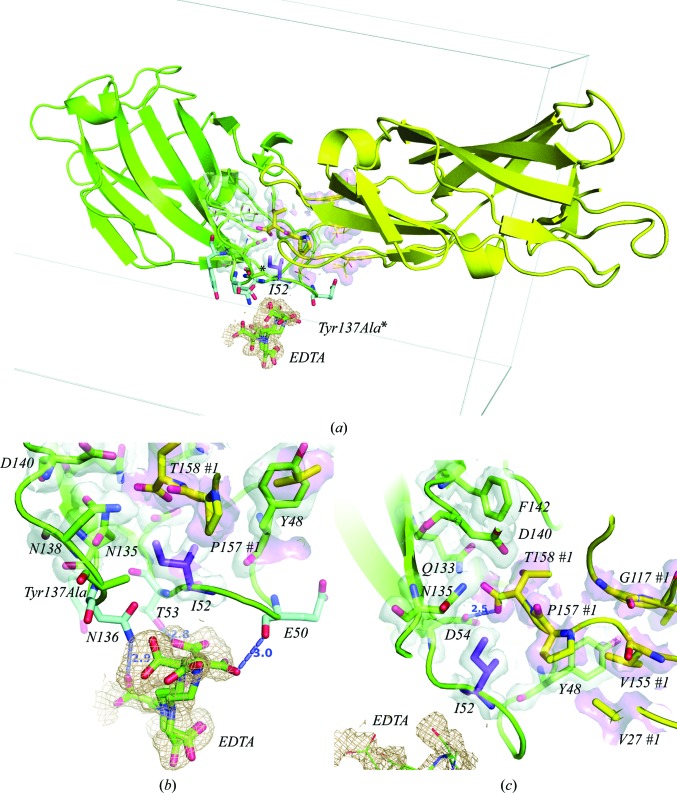
Crystal structure of the ligand-free Y137A FimH mutant. Electron density is displayed in white or light rose contourings at 1.5σ (2*F*_o_ − *DF*_c_) for residues of the molecule in the asymmetric unit (green) and for the neighbouring FimH (yellow), respectively. (*a*) Proximity of the mannose-binding Asp54 at the bottom of the pocket (shown as a green ball-and-stick model) to the EDTA-binding cavity. Ile52 is shown as a magenta ball-and-stick model. Ile52 couples the two sites displayed in more detail in (*b*) and (*c*). (*b*) The EDTA-binding cavity of ligand-free Y137A FimH, with hydrogen bonds from EDTA to Asn136, Thr53 and Glu50 shown as blue ball-and-stick models. (*c*) The mannose-binding pocket showing the strong hydrogen bond of Asp54 to the FimH lectin-domain C-­terminal carboxylate of Thr158 in the symmetry-related FimH molecule 1.

As a consequence of the Y137A mutation in FimH, the adjacent lectin domain plugs its polypeptide C-terminal end into the mannose-binding pocket of one FimH molecule (Fig. 3 ▸ *a*). The Val155-Val156-Pro157-Thr158 peptide of the FimH lectin domain from an adjacent symmetry-related lectin domain makes a significant intrusion where the oligomannoside glycan is normally bound (Wellens *et al.*, 2008 ▸). The carboxylate group of Thr158 makes a strong hydrogen-bond interaction (2.5 Å) with Asp54 at the bottom of the mannose-binding pocket (Fig. 3 ▸ *c*). In addition, an EDTA buffer molecule is bound in different alternate conformations into a cavity on the FimH surface. EDTA is held in place by means of multiple van der Waals interactions and potentially three strong hydrogen bonds made by Glu50 (backbone carbonyl at 3.0 Å), Thr53 (side-chain hydroxyl at 2.8 Å) and Asn136 (side-chain amide group at 2.9 Å) to three different carboxylate groups of EDTA (Fig. 3 ▸ *b*). Remarkably, EDTA was only contained in the extraction buffer of FimH extraction from the *E. coli* periplasm, but remained bound during purification and dialysis steps, which did not contain EDTA (see also §[Sec sec2]2). The EDTA binding site is very close to the mannose-binding pocket and the two sites are coupled by residue Ile52 (Fig. 3 ▸). The protein backbone holding the mutated residue 137 separates both sites (Fig. 3 ▸ *a*). The Y137A mutant structure allows the binding of a macromolecule other than mannose in the FimH mannose-binding pocket to be observed for the first time and moreover exposes a hitherto undiscovered adjacent binding pocket on the FimH surface.

### 3.4. Ligand binding in the crystal structures of FimH tyrosine-gate mutants   

**Figure 4:**
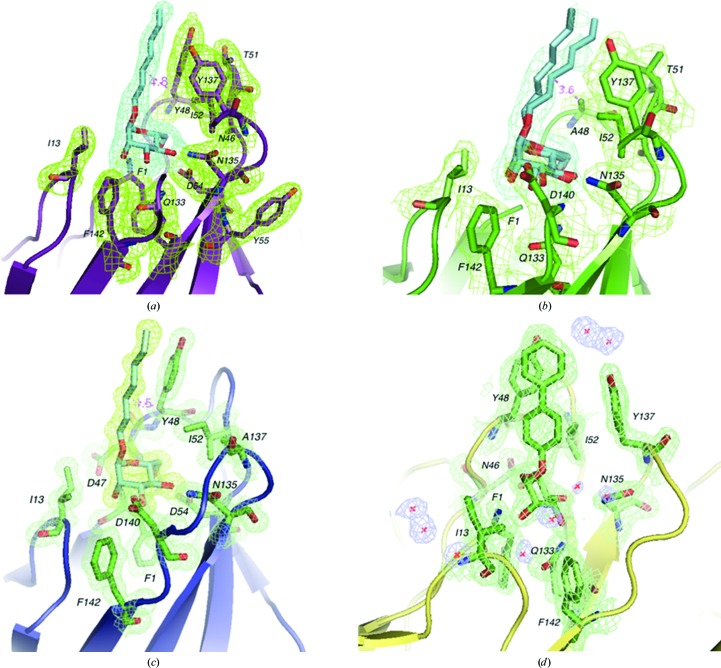
Mannose-binding sites of the crystal structures of FimH tyrosine-gate mutants and their complexes with HM and BF. Electron densities are shown at 1.5σ (green) and/or 0.8σ (light blue) (2*F*_o_ − *DF*_c_) for the protein and the mannosides and at 1.5σ (2*F*_o_ − *DF*_c_) (dark blue) for visible water molecules. The mannose-binding sites of the (*a*) WT FimH–HM, (*b*) Y48A FimH–HM, (*c*) Y137A FimH–HM and (*d*) WT FimH–BF complexes demonstrate a typical stacking pattern (see text for more details).

Insights into the molecular interactions of the Y48A and Y137A mutant FimH lectin domains with mannosides are obtained by solving their X-ray crystal structures either in the ligand-free form or in complex with the compounds HM or BF (Figs. 3 ▸ and 4 ▸ and Table 1 ▸). The two new tyrosine-gate mutant structures showed highly similar backbone conformations, with an r.m.s.d. for all C^α^ atoms of 0.402 Å for Y137A FimH and 0.368 Å for Y48A FimH relative to the wild-type structure (PDB entry 4buq), and a similar local binding-site conformations as in the wild type. The overall structure of the FimH lectin is thus not strongly impacted by the mutations, in agreement with the CD spectra and unfolding experiments (Fig. 1 ▸). The HM ligand is found to be similarly bound in the crystal structures of Y137A and WT FimH (Figs. 4 ▸ *a* and 3 ▸ *c*), despite the fact that the heptyl aglycone can interact with the aromatic side chains of both tyrosine-gate residues in WT FimH and only with Tyr48 in the Y137A mutant. The heptyl aglycone of HM demonstrates at least two different conformations in the Y48A mutant, of which one penetrates into the space of the missing Tyr48 side chain (Fig. 4 ▸ *b*), as observed by its shorter distance from the Ala48 mutant residue (3.6 Å instead of 4.5 and 4.8 Å in Y137A and WT FimH, respectively; Fig. 4 ▸). This disorder of the HM ligand in the Y48A crystals is congruent with a marked increase in entropic contribution to the interaction (Table 3 ▸). In stark contrast, the binding of BF to the Y48A mutant is not paired with an increase in entropy (Table 3 ▸). Despite the absence of a crystal structure to provide insight, the ITC data may indicate that the presence of the biphenyl aglycone on BF can provide enthalpy-driven interactions with Tyr137 and Ile52 to compensate for the lack of the Tyr48 aromatic ring. In the crystal structure of the Y137A FimH mutant in complex with HM the binding site is too involved in crystal packing to allow the observation of disorder in the ligand binding (Fig. 4 ▸ *c*).

The crystal structure of wild-type FimH in complex with BF demonstrates stacking of its aromatic biphenyl aglycone with both tyrosines of the gate (Fig. 4 ▸ *d*). BF shows the same binding mode, ligand conformation and crystal-packing environment in each of the eight FimH monomers, hovering over the closed tyrosine gate comprised of Tyr48 and Tyr137, similar to what is observed for the two FimH monomers co-crystallized with BF in a different space group (*P*2_1_2_1_2_1_; PDB entry 4x50; Fiege *et al.*, 2015 ▸). This same mechanism of compensation by aromatic ring substituents does not hold true for the Y137A mutant, where a large loss in affinity is experienced (Table 3 ▸). The lack of the Tyr137 side chain can thus not be compensated for, probably because of its larger distance from the mannose. It has been documented that the presence of the Tyr48 side chain hinders the fit of phenyl α-d-mannosidic compounds into the binding site, however with an almost perfect enthalpy–entropy compensation (Roos *et al.*, 2013 ▸). The steric hindrance by Tyr48 renders the binding highly dynamic, whereby the aromatic ligand seeks multiple stacking modes predominantly with the two tyrosines, thus including Tyr137 (de Ruyck *et al.*, 2016 ▸). In conclusion, the absence of the Tyr137 side chain provokes a more substantial loss of enthalpy-driven interactions than the absence of the Tyr48 side chain for both HM and BF.

The backbone dihedral angle values of Tyr48 and Tyr137 in the wild-type FimH lectin have always been intriguing, because their φ (torsion around the C^α^—N bond) and ψ (torsion around the C—C^α^ bond) dihedral angles are in a region of the Ramachandran plot that is not favoured but allowed (Supplementary Table S2). Remarkably, the φ/ψ-angle values of residues 48 and 137 in the alanine-mutant crystal structures solved here correspond to favoured backbone conformations (Supplementary Table S2). These findings, together with the fact that the crystal structures could not fully explain the ITC measurements, prompted us to investigate the behaviour of the backbone dihedral angles of the regions containing Tyr48 and Tyr137 by employing molecular simulation techniques.

### 3.5. The Y137A mutation relaxes the backbone dihedral angles   

**Figure 5:**
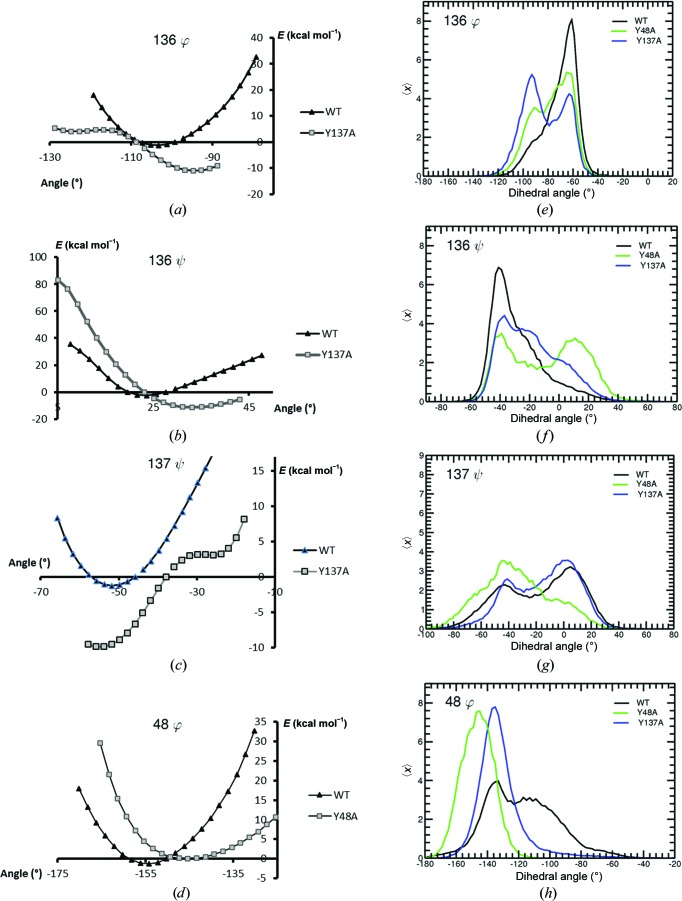
The effect of mutation on backbone dihedral angles. QM energy profiles are shown for the (*a*) 136 φ, (*b*) 136 ψ, (*c*) 137 ψ and (*d*) 48 φ dihedral angles. The energy associated with the dihedral angle found in the crystal structure is used as a reference (0 kcal mol^−1^ on the vertical axis). Additionally, MD probability distributions are shown for (*e*) 136 φ, (*f*) 136 ψ, (*g*) 137 ψ and (*h*) 48 φ dihedral angles as extracted from 3 × 50 ns MD trajectories of ligand-free wild-type (black), Y48A (green) and Y137A (blue) FimH. For each angle, the probability 〈*x*〉 of finding the dihedral angles at a certain value is plotted.

The flexibility of the amino-acid regions 47–49 and 136–138 was assessed using QM calculations in ligand-free FimH by calculating the relative energies related to the rotation of the backbone dihedral angles φ and ψ of a five-residue peptide centred either around Tyr48 or Tyr137. These relative energies of the φ and ψ angles upon rotation are plotted for Y137A and Y48A, using the energy of the angle found in the crystal structure as a reference, and compared with those of the WT FimH protein (Supplementary Table S2 and Figs. 5 ▸ *a*–5 ▸ *d*). Also, the backbone dihedral angle distributions of the corresponding residues were extracted from the MD trajectories and analysed (Figs. 5 ▸ *e*–5 ▸ *h*). As no crystal structures carrying either the Y48A or Y137A mutation were available for BF, we generated all MD simulations from the WT structures after introducing the mutation *in silico* (see §[Sec sec2.11]2.11). In addition, MD simulations were carried out on the available ligand-free and HM-bound mutated crystal structures (see §[Sec sec2.11]2.11). In all MD trajectories the backbone r.m.s.d. and secondary-structure content is comparable (Supplementary Tables S3 and S4) and are in good agreement with an earlier MD simulation on WT FimH (Singa­ravelu *et al.*, 2014 ▸). This finding prompted us to use the trajectories of proteins featuring *in silico* mutations for further analysis.

The relative QM energy plots show that the mutation of Tyr137 to alanine results in a relaxation of the backbone dihedral angles of residue 137 and of the adjacent Asn136 (Figs. 5 ▸ *a*, 5 ▸ *b* and 5 ▸ *c*). Rotating around these dihedral angles in the Y137A mutant FimH causes a much smaller energy penalty compared with WT FimH, thus allowing these dihedral angles to sample a much broader range of values. The dihedral angle distribution extracted from the MD trajectories shows a similarly increased flexibility, allowing a wider sampling range of the dihedral angle values of Asn136 in the Y137A mutant compared with WT FimH (Figs. 5 ▸ *e*, 5 ▸ *f* and 5 ▸ *g*).

For the Y48A FimH, the mutation does not alter the shape of the energy curve in all but one case (Supplementary Fig. S4): the φ angle of residue 48 (Fig. 5 ▸ *d*; to a smaller extent, the ψ dihedral angle curve of residue 49 also is flatter; see Supplementary Fig. S4*a*). Its rotation to a higher dihedral angle value causes a less severe energy penalty compared with wild-type FimH. These results largely agree with the dihedral angle distribution obtained from the MD trajectories (Supplementary Fig. S5), showing that the mutation of Tyr48 impacts the distribution of the Asp47 φ/ψ and the Tyr48 φ/ψ dihedral angles (Fig. 5 ▸ *h* and Supplementary Fig. S5), while leaving the other backbone dihedral angles unchanged in this region. It appears that the residues surrounding Tyr48 stabilize its position and more greatly restrict its freedom to change conformation, compared with Tyr137 that is located in a loop near the protein surface (Fig. 3 ▸ *a*).

To summarize, the Y137A mutant samples a wider range of dihedral angles with lower energy than WT FimH, whereas at position 48 the sampling range of dihedral angles is restricted upon mutation to alanine. The energy level of the Y48A mutant is less impacted compared with the WT, congruent with the low impact of this mutation on the affinity of FimH towards BF and HM (Table 3 ▸).

### 3.6. Backbone dynamics of Tyr48 and Tyr137 are coupled by the mannose-binding residue Ile52   

**Figure 6:**
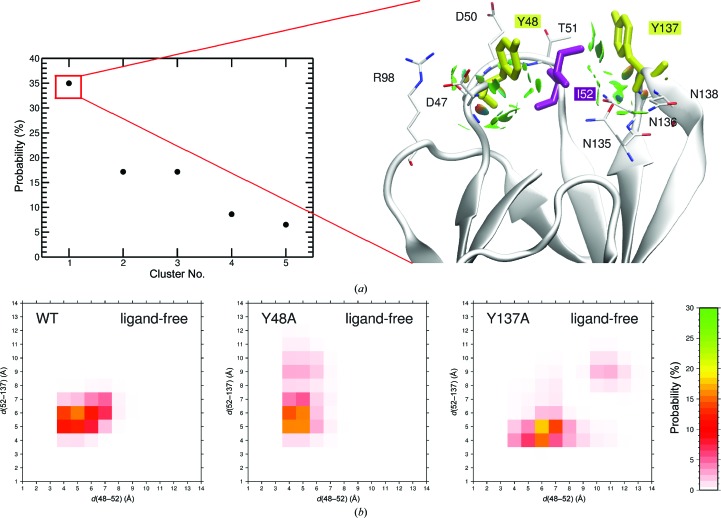
The motions of Tyr48 and Tyr137 are coupled, mediated by Ile52. (*a*) The five major clusters of the tyrosine gate are shown extracted from the MD trajectories of ligand-free WT FimH (left). Clusters featuring similar structures were extracted from the 150 ns simulations and plotted against their population occurrence (in %). Only the five most abundant clusters, together counting for more than 80% of all possible conformations, are shown. For the cluster with the highest occurrence, the interactions between the two tyrosine residues and all other protein residues are also shown (right). (*b*) Probability of having the centre of mass of the side chain of Ile52 at a certain distance from the centre of mass of the Tyr48 and the Tyr137 side chain at the same time. The probabilities are shown for the ligand-free simulations of the WT (left), the Y48A mutant (middle) and the Y137A mutant (right). In the WT Tyr48 and Tyr137 show a clear preference for being close to Ile52 (a distance of the two centres of mass of <6 Å), indicating an Ile52-mediated coupling of the motion in the tyrosine gate. This connection is weakened (Y48A) or lost (Y137A) following mutation of these residues.

The analysis of the MD trajectories highlights that the Y137A mutation impacts the φ/ψ dihedral angle of Tyr48 on the opposite side of the tyrosine gate, while leaving the other backbone dihedral angles in the region 47–49 unchanged (Fig. 5 ▸ *h* and Supplementary Fig. S5*b*). In addition, the Y48A mutation also impacts the φ/ψ dihedral angles of residue 136 and the ψ angle of Tyr137 (Supplementary Fig. S5*a*). This can be explained if the motions of Tyr48 and Tyr137 are connected to each other. Indeed, in the most populated Tyr48 and Tyr137 configurations (Fig. 6 ▸ *a*) both aromatic rings interact with Ile52, through which a connection between the two tyrosines can be established. The probabilities that the centre of mass between Ile52 and Tyr48 and between Ile52 and Tyr137 are at a certain distance (Fig. 6 ▸ *b*), analysed on the full-length WT trajectories, further highlights an Ile52-mediated coupling of the motion of Tyr48 and Tyr137. Ile52 has previously been identified to make hydrophobic contacts with the C2—C3 bond of bound α-d-mannose (Gouin *et al.*, 2014 ▸).

The interaction between Tyr137 and Ile52 (in Y48A) and between Tyr48 and Ile52 (in Y137A) is weakened by the mutations (Fig. 6 ▸ *b*), and the remaining tyrosine residue of the gate is liberated to move freely. The weakening is more pronounced for the Y137A mutation. The Ile52-mediated connection might be needed in WT FimH for proper guidance of the ligand to its binding site. Ile52 is also the residue directly linking the protein-intruded mannose-binding site inside the Y137A mutant FimH, with the EDTA binding site formed by Asn136, Thr53 and Glu50 at the protein surface (Fig. 3 ▸).

### 3.7. The ligand-free state of Y137A FimH samples backbone dihedral angles of the mannose-bound state of wild-type FimH   

**Figure 7:**
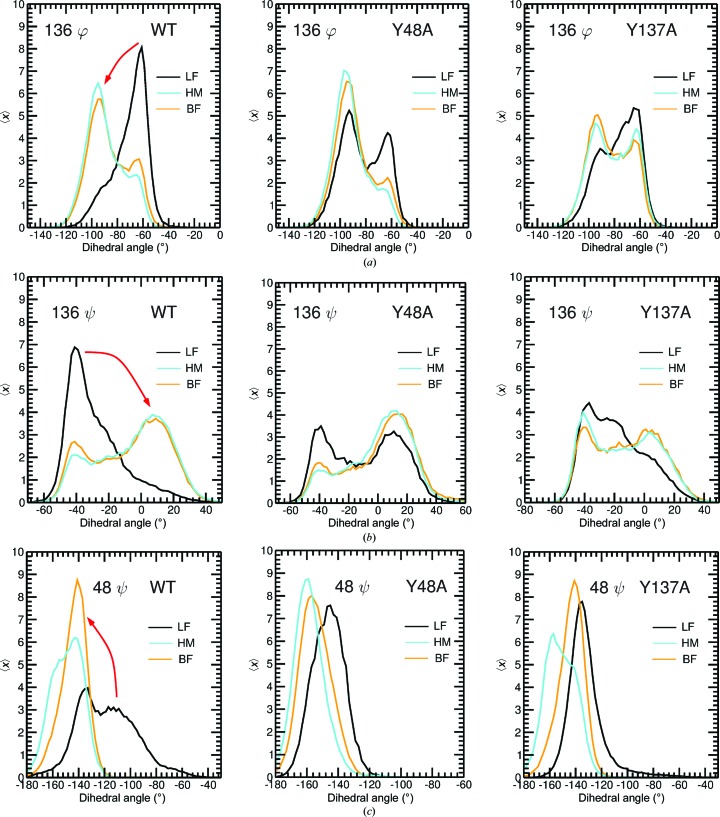
Backbone dihedral angle distributions as extracted from ligand-free and ligand-bound MD simulations of WT FimH and the Y48A and Y137A mutants. The probability 〈*x*〉 of finding the (*a*) 136 φ, (*b*) 136 ψ and (*c*) 48 φ dihedral angles at a certain value is plotted as extracted from ligand-free (LF) simulations (black) or simulations with HM (cyan) or BF (orange) bound to WT (left), Y48A (middle) and Y137A (right) FimH. The distributions were calculated over a total of 3 × 50 ns MD trajectories. The arrows indicate the conformational change upon ligand binding, which is only observed in the WT.

The backbone φ/ψ dihedral angle distributions of most of the residues in the 47–49 and 136–138 regions in wild-type FimH protein show a shift in the maximum upon the binding of HM or BF (Fig. 7 ▸ and Supplementary Figs. S6 and S7). This shift is lost in the Y137A mutant for most of the dihedral angles in the region of residues 136–138 (Fig. 7 ▸ and Supplementary Figs. S6 and S7). In Y137A, the backbone dihedral angle distributions of residues 136–138 in the unbound state already resemble the distributions in the bound state. In the Y48A mutant, the backbone dihedral angle distributions of residues 47 and 48, but not of Pro49, undergo a change (Supplementary Fig. S7). The effect of the mutation is less pronounced for the Y48A mutant compared with the Y137A FimH, in which almost all of the angles were impacted (compare Supplementary Figs. S6 and S7).

This indicates that in wild-type FimH the 136–138 region undergoes a conformational change upon ligand binding, whereas in the Y137A mutant no conformational change takes place. Such a conformational change can lead to a loss of energy that normally would go into the binding of the ligand. It can be hypothesized that the high energy and geometric stringency inherent to the binding pocket of wild-type FimH has been optimized to enhance interactions with the ligands and to direct selective and specific binding with high affinity (Table 3 ▸).

### 3.8. Ligand dynamics are differently impacted by the tyrosine-gate mutations   

**Figure 8:**
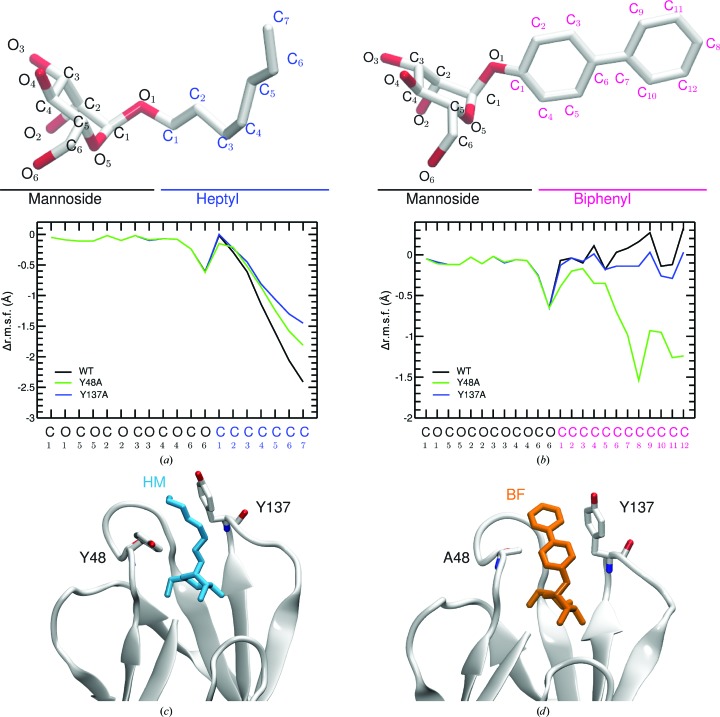
Flexibility of ligands bound to WT FimH and the Y48A and Y137A mutants compared with the situation in water alone. The difference in flexibility for the WT (black), Y48A (green) and Y137A (blue) FimH MD trajectories (given as the Δr.m.s.f.) is plotted for the ligands (*a*) HM and (*b*) BF against the heavy-atom name of the ligand atoms. In addition, the ligand is depicted with atom names above each plot. The most representative conformation of (*c*) the HM ligand (cyan) and (*d*) the BF ligand (orange) is depicted for the trajectories with the lowest Δr.m.s.f. values (HM, WT; BF, Y48A). The protein is shown as a white cartoon; residues 48 and 137 are shown as ball-and-stick models and coloured atomwise.

As the binding might also impact the flexibility of the ligand, we computed the difference in flexibility of the ligand in water and in the protein (Fig. 8 ▸). The binding of HM to wild-type FimH largely restricts its flexibility (Fig. 8 ▸ *a*). The restriction is a little less pronounced in the Y48A mutant and is even further softened in the Y137A mutant. In contrast to HM, the flexibility of BF is almost not restricted in the WT binding site and also not in the Y137A mutant. In the case of the Y48A mutant the flexibility of BF is largely restricted compared with its flexibility in water (Fig. 8 ▸ *b*). The largest decrease is found for the second aromatic ring of the BF aglycon part. This lower flexibility arises from a T-shaped π–π stacking between the second aromatic ring of BF and the Tyr137 aromatic ring observed in most of the Y48A MD simulation snapshots (Fig. 8 ▸ *c*) that is absent in the wild-type and Y137A MD simulations. This stacking could not have been predicted from the crystal structure of WT FimH in complex with BF, which shows aromatic stacking with Tyr48 only (Fig. 4 ▸ *d*), but it may explain why there is such a little loss in the enthalpic contribution for BF binding in Y48A FimH (Table 3 ▸).

### 3.9. Molecular effect of the mutations on HM and BF binding   

The objective of this study is to assess the contributions of the tyrosine-gate residues to the binding of mannosides to the FimH lectin domain. We have therefore generated two single-residue mutants, Y48A and Y137A, in FimH. No significant global secondary-structure differences are shown by CD and unfolding experiments, or in the crystal structures and MD simulations. However, the binding affinities obtained by a competition assay and a thermodynamic assay show a large impact of the Y137A mutation, whereas the affinities are only minimally affected for the Y48A mutation. Interactions and dynamics are related to the enthalpic and entropic contributions to the reaction, respectively. The Y48A mutation has some, but a smaller, effect on the different studied parameters and the ensemble adjusts more easily to these changes, in accordance with the enthalpy–entropy compensation seen in ITC measurements. The thermodynamic fingerprints of the Y137A, on the other hand, showed reduced enthalpic contributions that are only partially compensated by enhanced entropy contributions (Table 3 ▸). The impact of the Y137A mutation cannot be remediated and thus the affinity of FimH is decreased.

**Figure 9:**
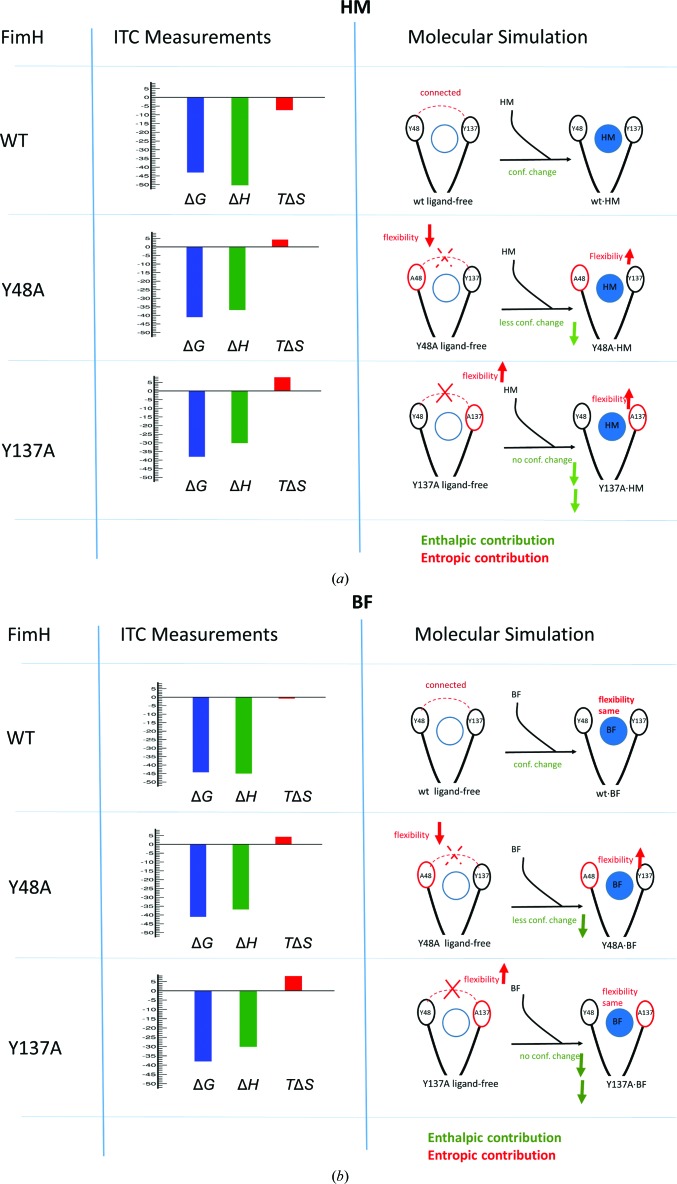
The different processes identified to play a role in the change in FimH affinity for binding (*a*) HM and (*b*) BF upon mutation of one of the tyrosine-gate residues. Both the results from the ITC measurements (Table 3 ▸) and from the molecular simulation (MD and QM; see §§3.5–3.8 [Sec sec3.6] [Sec sec3.7] [Sec sec3.8]) are summarized.

The crystal structures indicate changes in the backbone dihedral angles owing to the mutation, which are confirmed by QM and MD simulations. A summary of the different processes is given in Fig. 9 ▸: (i) in the ligand-free state the dynamics of the 136–138 region are increased owing to the Y137A mutation; (ii) in contrast the flexibility of the 47–49 region is reduced (MD data) or unchanged (QM) in the case of the Y48A FimH mutant; (iii) the motions of Tyr137 and Tyr48 are coupled by Ile52, and each of these residues can move more freely after mutation of the other; (iv) a conformational change of the 136–138 region takes place in wild-type FimH upon binding of the ligand and this change is largely lost in the Y137A mutant, but only slightly in the Y48A mutant; (v) the dynamics of the HM ligand are restricted upon binding to wild-type FimH and both tyrosine mutations increase the flexibility of HM in the binding site and, finally, (vi) such a restriction is not observed for the binding of the BF ligand to wild-type FimH; however, in the Y48A FimH mutant a decrease of the dynamic behaviour of BF is observed in the binding site through the formation of compensating inter­actions (Figs. 8 ▸ *b* and 8 ▸ *d*).

In conclusion, this study highlights the need to combine several theoretical and experimental methods to elucidate the molecular mechanisms of the changes in binding affinity of FimH upon mutation and can be seen as a case study for further investigations.

## 4. Conclusion   

In this work, we present novel crystal structures of the FimH lectin with mutations in the tyrosine gate, namely of tyrosines 48 or 137 to alanine. The mutations have a moderate effect on the affinity of FimH for mannose, but a markedly lower affinity is observed for heptyl- and biphenyl-substituted mannosides that intercalate in the tyrosine gate. In the crystals of the Y137A mutant, a breakdown of the binding site with a severe loss of specificity is observed. Using quantum-mechanical calculations, we could demonstrate that the wild-type Tyr137 introduces strain in the polypeptide backbone. This maintains a high energetic potential that is normally only released upon the binding of an oligomannosidic ligand. Using molecular-dynamics simulations, we could highlight that this energetic potential is coupled to the other tyrosine of the gate, Tyr48, *via* the inner mannose-binding residue Ile52. In conclusion, the mutation of Tyr137 to alanine relaxes the binding site prematurely, whereby the stringent selectivity of the FimH lectin for mannose is disrupted and the binding affinity decreases.

## Supplementary Material

PDB reference: FimH, wild type, complex with HM, 4buq

PDB reference: Y48A mutant, complex with HM, 4ca4

PDB reference: Y137A mutant, complex with HM, 5fs5

PDB reference: wild type, complex with BF, 5fwr

PDB reference: Y137A mutant, 5fx3

Supporting information.. DOI: 10.1107/S2052252516016675/jt5015sup1.pdf

## Figures and Tables

**Table 1:** Crystallization conditions, data collection, refinement statistics and model geometry Values in parentheses are for the outer shell.

	WT FimH–HM	Y48A FimH–HM	Y137A FimH–HM	WT FimH–BF	Y137A FimH
Crystallization conditions	5%(*w*/*v*) PGA-LM†, 0.1 *M* Tris–HCl pH 7.8, 30%(*v*/*v*) PEG 550 MME	5%(*w*/*v*) PGA-LM, 100 m*M* sodium cacodylate pH 6.5, 200 m*M* MgCl_2_	5%(*w*/*v*) PGA-LM, 100 m*M* sodium cacodylate pH 6.5, 12%(*w*/*v*) PEG 8K	5%(*w*/*v*) PGA-LM, 100 m*M* Tris pH 7.8, 20%(*w*/*v*) PEG 3350	0.2 *M* sodium malonate, 0.1 *M* bis-tris propane pH 7.5, 20%(*w*/*v*) PEG 3350
Ligand	10 m*M* *n*-heptyl α-D-mannopyranoside	10 m*M* *n*-heptyl α-D-mannopyranoside	10 m*M* *n*-heptyl α-D-mannopyranoside	5 m*M* 4-biphenyl α-D-mannopyranoside	—
Data collection					
Wavelength (Å)	0.918410	0.98011	0.918410	0.918410	0.98011
Beamline	PX14.2	PROXIMA 1	PX14.2	PX14.2	PROXIMA 1
Synchrotron	BESSY II	SOLEIL	BESSY II	BESSY II	SOLEIL
Resolution range (Å)	45–2.20	41–2.84	15–1.40	48–2.13	46.92–1.80
No. of reflections	146524	65358	170620	134828	225919
No. of unique reflections	20238	9163	25691	85046	21963
〈*I*/σ(*I*)〉	12.33 (3.77)	8.58 (3.02)	17.03 (4.15)	9.07 (3.09)	13.27 (2.80)
Completeness (%)	98.0 (90.8)	97.7 (99.1)	98.4 (91.8)	93.8 (84.2)	99.6 (99.7)
Crystal mosaicity (°)	0.448	0.111	0.445	0.540	0.546
*R*_meas_ ‡ (%)	13.3 (57.1)	22.0 (81.1)	9.1 (47.4)	11.3 (43.2)	14.9 (146.9)
Wilson *B* factor (Å^2^)	35.9	43.1	8.6	20.3	15.3
Space group	*P*2_1_2_1_2_1_	*P*4_3_2_1_2	*P*22_1_2_1_	*P*1	*P*6_5_22
No. of molecules in asymmetric unit	2	2	1	8	1
Unit-cell parameters					
*a* (Å)	60.30	89.85	45.97	53.02	54.18
*b* (Å)	68.07	89.85	59.55	74.00	54.18
*c* (Å)	95.61	91.87	96.88	111.77	257.95
α (°)				99.32	90
β (°)				102.97	90
γ (°)				97.83	120
Refinement					
*R*_work_	0.205	0.179	0.149	0.224	0.177
*R*_free_ (test set = 5%)	0.256	0.235	0.183	0.333	0.217
R.m.s.d.s and stereochemistry					
R.m.s.d., bonds (Å)	0.008	0.015	0.006	0.015	0.010
R.m.s.d., angles (°)	1.098	1.712	1.232	1.287	1.111
Ramachandran plot, residues (%)					
Favoured region	96.83	98.00	96.20	93.51	97.00
Allowed region	3.17	2.00	3.80	6.33	3.00
Outliers	0.0	0.0	0.0	0.16	0.0
PDB entry	4buq	4ca4	5fs5	5fwr	5fx3

† Poly-γ-glutamic acid 200–400 kDa low-molecular-weight polymer.

‡ *R*_meas_ is the redundancy-independent merging *R* factor (Karplus & Diederichs, 2015 ▸).

**Table 2:** Stability and folding cooperativity of FimH tyrosine-gate mutants as derived from GdmCl-induced unfolding (Fig. 1 ▸ *c*) All energies are given in kJ mol^−1^.

	WT FimH	Y48A FimH	Y137A FimH
Free energy of folding	−50.00 ± 3.07	−44.05 ± 2.37	−53.71 ± 4.53
Folding cooperativity	18.17 ± 1.11	15.95 ± 0.85	19.63 ± 1.64

**Table 3:** Thermodynamic fingerprints of the binding of mannosides to WT, Y48A mutant and Y137A mutant FimH lectin domains

Ligand	FimH lectin	*N*	Δ*G* (kJ mol^−1^)	Δ*H* (kJ mol^−1^)	*T*Δ*S* (kJ mol^−1^)	*K*_d_ (n*M*)	r*K*_d_	rIC_50_
AM	WT	1.03	−34.0	−43.1	−9.1	1125	1	1
	Y48A	0.96	−31.4	−40.6	−9.2	2715	2.4	1.4
	Y137A	0.98	−31.8	−39.7	−7.9	2735	2.4	1.2
HM	WT	0.98	−43.0	−50.3	−7.3	28.9	1	1
	Y48A	1.02	−41.0	−36.8	4.2	65.5	2.3	1.3
	Y137A	1.00	−38.0	−30.1	7.9	206.4	7.1	8.7
BF†	WT	1.07	−44.2	–45.0	−0.8	17.7	1	1
	Y48A	1.06	−41.8	−42.2	−0.4	46.5	2.6	0.7
	Y137A	1.04	−40.1	−35.5	4.6	89.7	5.1	5.8

† 2.5% DMSO was added to keep BF soluble (Fiege *et al.*, 2015 ▸).
