# Supplementary material for: Mutation of Tyr137 of the universal Escherichia coli fimbrial adhesin FimH relaxes the tyrosine gate prior to mannose binding
Source: IUCrJ. 2017 Jan 1;4(Pt 1):7–23. doi: 10.1107/S2052252516016675 (PMC5331462; doi:10.1107/S2052252516016675)
Supplement: Supplementary file 1 [file m-04-00007-sup1.pdf]

IUCrJ

Volume 4 (2017)

Supporting information for article:

Mutation of Tyr137 of the universal *Escherichia coli* fimbrial adhesin FimH relaxes the tyrosine gate prior to mannose binding

Said Rabbani, Eva-Maria Krammer, Goedele Roos, Adam Zalewski, Roland Preston, Sameh Eid, Pascal Zihlmann, Martine Prévost, Marc F. Lensink, Andrew Thompson, Beat Ernst and Julie Bouckaert

Table S1 Thermodynamic measurements of **AM**, **BF** and **HM** binding to the lectin domain of WT FimH and Y48A and Y137A mutants using isothermal titration calorimetry (ITC).

Confidence intervals are given between parentheses. In the white rows are represented measurements repeated with different c-values.

**Cpd**	**Protein**	**Ligand / Protein conc. [µM]**	**K_D [nM]**	**$\Delta G°$ [kJ/mol]**	**$\Delta H°$ [kJ/mol]**	**$-T\Delta S°$ [kJ/mol]**	***N***	**c-value**
**AM**	**WT**	400 / 27.0	1125 (980 - 1292)	-34.0	-43.1 (-42.1 - -43.8)	9.1	1.03	24
**AM**	**Y48A**	300/ 15.5	2715 (2403 - 3075)	-31.8	-40.8 (-39.3 - -42.4)	9.0	0.96	6
**AM**	**Y137A**	450 / 21.5	2735 (2393 - 3125)	-31.8	-39.7 (-38.3 - -41.1)	7.9	0.98	8
HM	WT	100 / 10.0	29.1 (25.7 - 32.9)	-43.0	-49.8 (-49.4 - -50.3)	6.8	0.98	343 (1)
HM	WT	100 / 8.6	29.0 (26.7 - 31.4)	-43.0	-50.9 (-50.5 - -51.2)	7.8	1.01	296
**HM**	**WT**		28.9 (25.8 - 32.3)	-43.0	-50.3 (-50.2 - -50.7)	7.3		
**HM**	**Y48A**	160 / 10.5	65.5 (53.2 - 80.0)	-41.0	-36.6 (-36.0 - -37.3)	-4.4	1.02	160
**HM**	**Y137A**	180 / 17.25	206.4 (184.0 - 230.5)	-38.2	-30.2 (-29.8 - -30.6)	-8.0	1.00	84
BF	WT	150 / 14.0	14.8 (13.9 - 15.9)	-44.7	-43.9 (-43.8 - -44.0)	-0.8	1.09	943
BF	WT	100 / 10.5	21.1 (16.7 - 25.9)	-43.8	-46.2 (-45.6 - -46.8)	2.4	1.06	500
**BF**	**WT**		17.7 (14.1 - 22.3)	-44.2	-45.0 (44.5 - -45.6)	0.8		
**BF**	**Y48A**	120 / 13.3	46.5 (37.1 - 57.6)	-41.9	-42.1 (-41.2 - -42.9)	0.2	1.06	286
**BF**	**Y137A**	220 / 14.3	89.7 (79.0 – 101.4)	-40.2	-35.5 (-35.1 - -35.8)	-4.8	1.04	159

Table S2 Backbone dihedral angles, φ and ψ, of the mutated tyrosines and 2 residues on each side were extracted using the program Molprobity for chain A from the different crystal structures: WT with bound HM (PDB entry 4BUQ), Y48A with bound HM (PDB entry 4CA4), Y137A with bound HM (PDB entry 5FS4), WT with bound BF (PDB entry 5FWR), and Y137A ligand-free (PDB entry 5FX3).

Crystal structure	Dihedral angle	Residues **46 - 50**				
		Asn46	Asp47	Tyr48	Pro49	Glu50
WT HM	φ	-95.1	-83.8	-150.2	-73.7	-61.1
	ψ	129.2	-11.0	56.1	-13.1	-31.4
Y48A HM	φ	-91.0	-98.2	-145.3	-62.5	-72.2
	ψ	137.0	-8.5	53.9	-22.1	-22.9
Y137A HM	φ	-101.4	-88.2	-149.1	-70.7	-59.5
	ψ	129.9	-8.0	57.6	-16.5	-26.8
WT BF	φ	-98.0	-76.5	-150.3	-66.7	-60.6
	ψ	124.2	-13.1	59.1	-17.1	-30.2
Y137A ligand-free	φ	-97.1	-75.7	-137.7	-68.1	-71.2
	ψ	123.1	-23.1	63.0	-23.8	-12.1
Crystal structure	Dihedral angle	Residues **135-139**				
		Asn135	Asn136	Tyr137	Asn138	Ser139
WT HM	φ	-148.7	-99.0	-123.2	-145.8	-98.9
	ψ	178.3	27.4	-45.8	-152.0	7.0
Y48A HM	φ	-162.0	-95.6	-106.4	-155.4	-58.2
	ψ	178.5	7.8	-37.5	-172.5	-21.9
Y137A HM	φ	-150.2	-107.9	-121.5	-153.3	-86.3
	ψ	-175.7	23.8	-37.2	-165.7	1.1
WT BF	φ	-150.0	-102.0	-133.0	-139.8	-97.3
	ψ	-176.8	33.0	-49.2	-162.9	27.9
Y137A ligand-free	φ	-151.9	-96.4	-112.4	-147.0	-98.4
	ψ	176.0	8.1	-34.8	-167.4	5.3

Allowed - Favored as pre-proline – Favored as *trans* proline – Favored

Table S3 Average RMSD of the protein backbone (C, N, O, and Cα atoms) compared to the starting point of each simulation is shown for all performed MD trajectories (for more details see Material and Methods).

The RMSD is given in Å with its standard deviation.

A) *in-silico* generated mutants

bound compound	Y48A	Y137A
-	1.0 ± 0.1	1.0 ± 0.1
**HM**	1.0 ± 0.1	1.1 ± 0.3
**BF**	1.0 ± 0.2	1.0 ± 0.2

B) available crystal structures

bound compound	WT	Y48A	Y137A
-	1.1 ± 0.2	-	1.1 ± 0.2
**HM**	1.1 ± 0.2	1.0 ± 0.1	0.9 ± 0.2
**BF**	1.2 ± 0.3	-	-

Table S4 Secondary structure content of the different MD simulations.

For all simulations the percentage of coiled/beta sheet /turn structures are given after averaging it over the simulations.

A) *in-silico* generated mutants

bound compound	Y48A	Y137A
-	19/52/24	19/52/24
**HM**	18/52/25	18/52/26
**BF**	18/51/26	18/52/25

B) available crystal structures

bound compound	WT	Y48A	Y137A
-	19/51/26	-	19/51/25
**HM**	18/51/26	19/52/25	18/50/26
**BF**	18/51/26	-	-

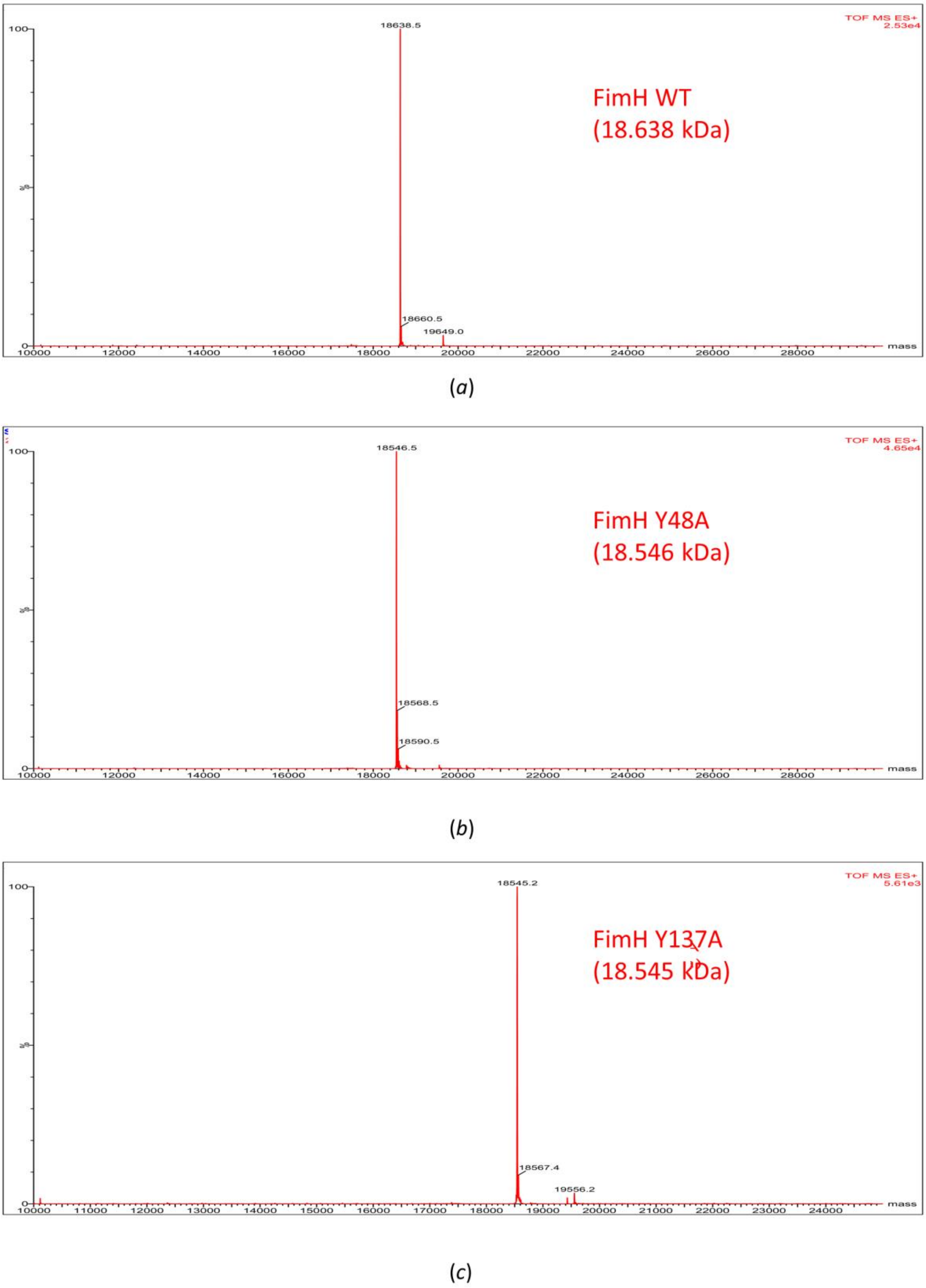

Figure S1 ESI-MS analysis to determine the exact molecular weight of (*a*) WT (18.638 kDa), (*b*) Y84A (18.546 kDa) and (*c*) Y137A FimH (18.545 kDa).

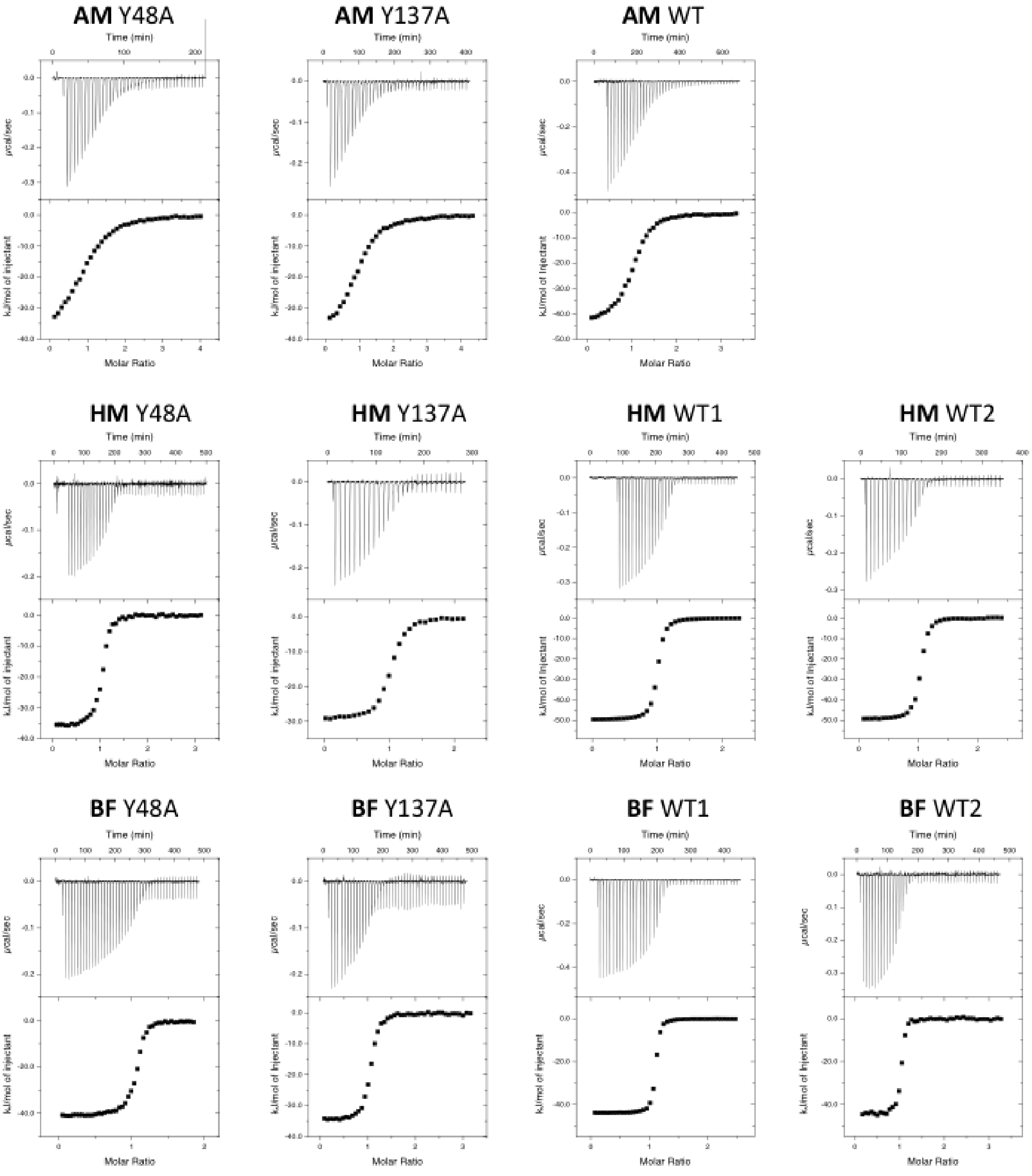

Figure S2 Enthalpograms of ligand **AM**, **HM** and **BF** binding to FimH. Repeated measurements to ensure accuracy of the high-affinity interactions of wild-type FimH are indicated as WT1 and WT2.

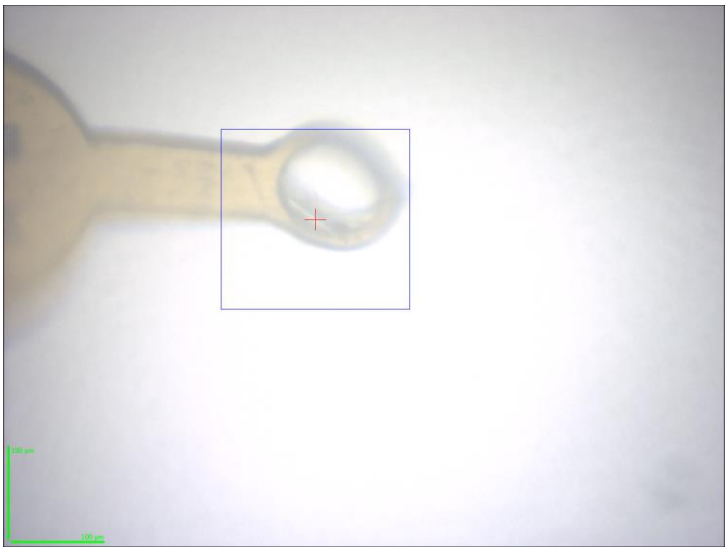

(*a*)

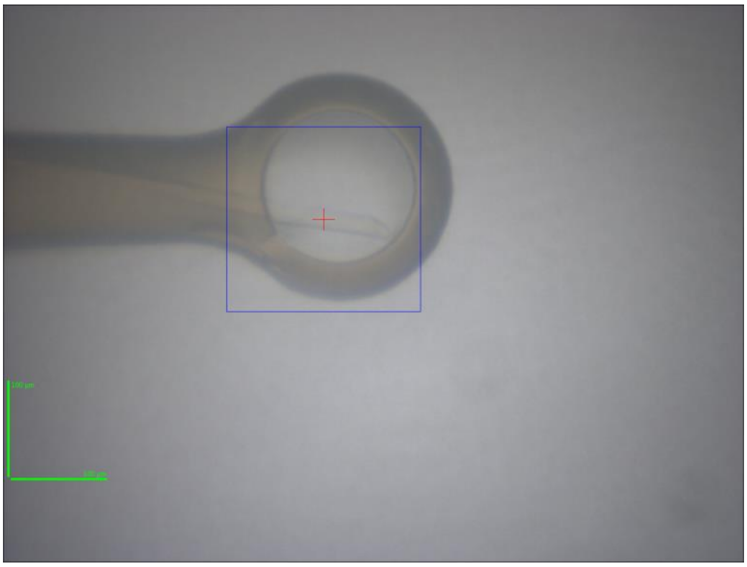

(*b*)

Figure S3 (*a*) Crystals of the Y48A FimH - HM complex. The crystal was only about 50 μm x 5 μm x 5 μm in size. (*b*) Crystals of ligand-free Y137A FimH. Tiny green scale bars measure 100 μm.

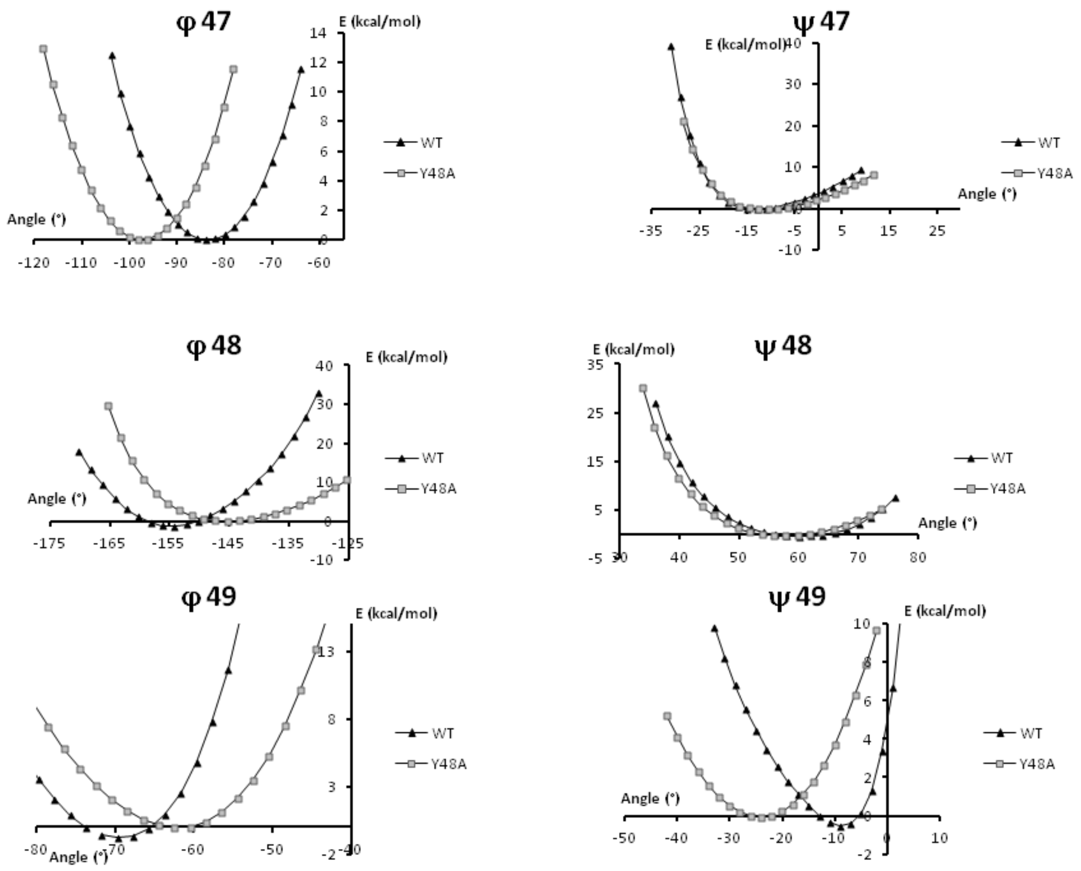

(*a*)

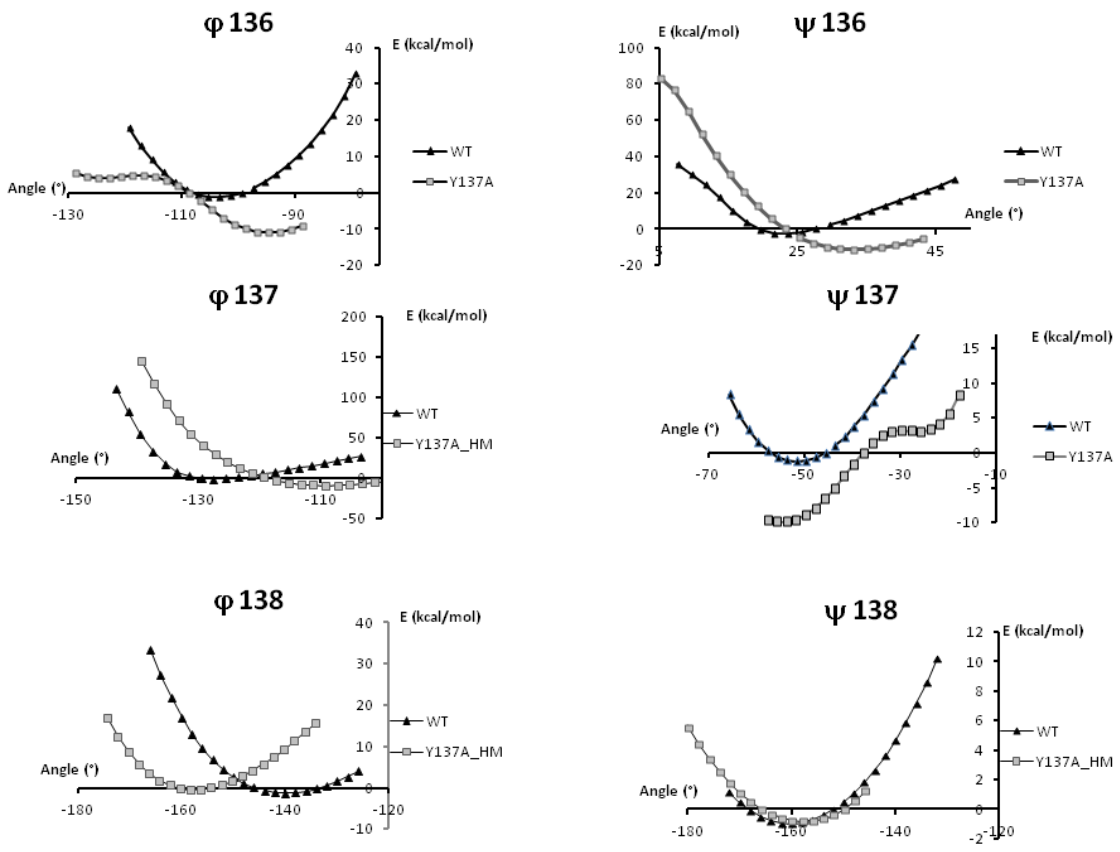

(*b*)

Figure S4 QM energy profiles showing the mutation effect on backbone dihedral angles. The energy profiles are shown (*a*) for the 47 to 49 and (*b*) for the 136 to 138 region. The energy associated to the dihedral angle found in the crystal structure is used as a reference (0 kcal/mol on the vertical axis).

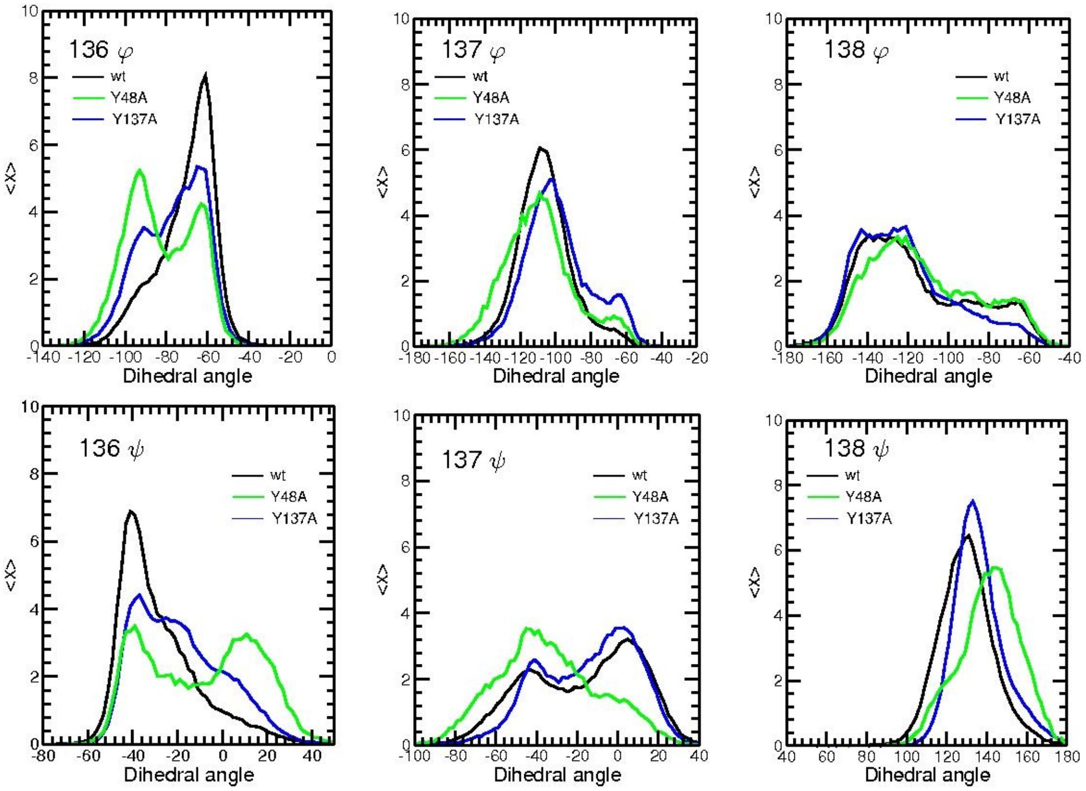

(*a*)

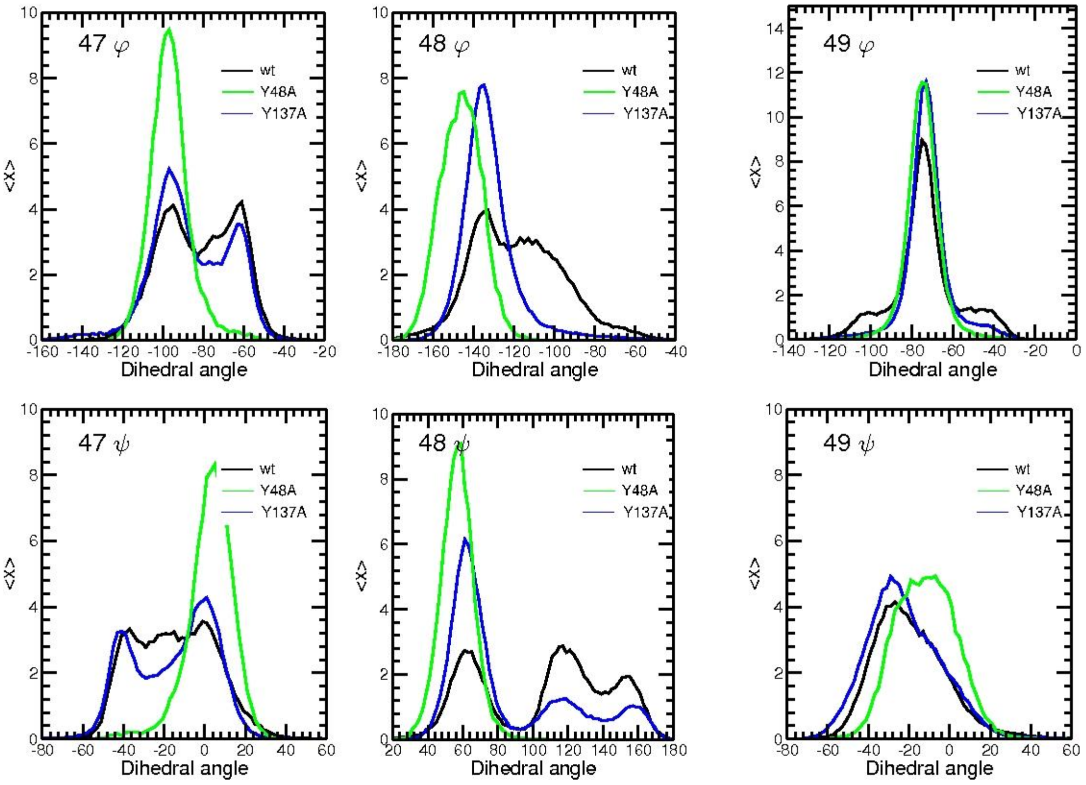

(*b*)

Figure S5 Backbone dihedral angle distributions as extracted from MD simulations of ligand-free WT, Y48A and Y137A FimH. The probability <x> of finding the backbone dihedral angles φ and ψ of

the peptides carrying residues (*a*) 136 to 138 and (*b*) 47 to 49, respectively, at a certain value is plotted as extracted from ligand-free WT (black), the Tyr48Ala (green) and the Tyr137A (blue) mutant simulations. The distributions are calculated over the total of 3x50 ns trajectories.

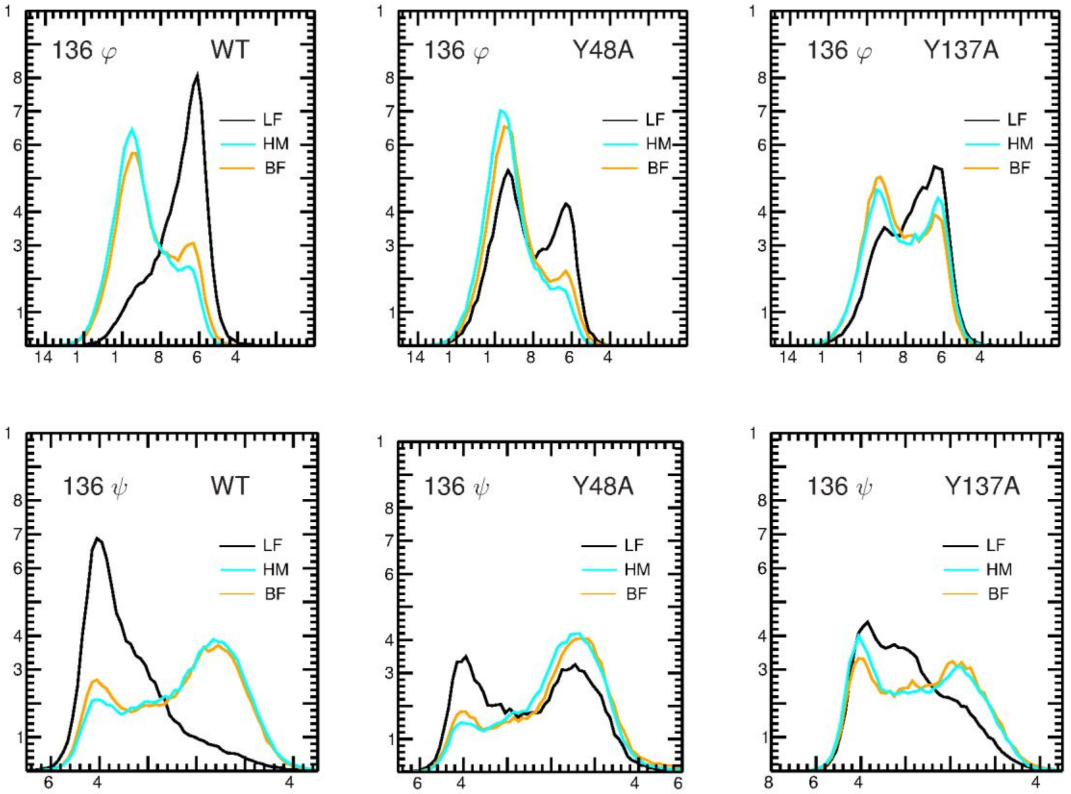
136 φ WT
136 φ Y48A
136 φ Y137A
136 ψ WT
136 ψ Y48A
136 ψ Y137A
LF
HM
BF

(a)

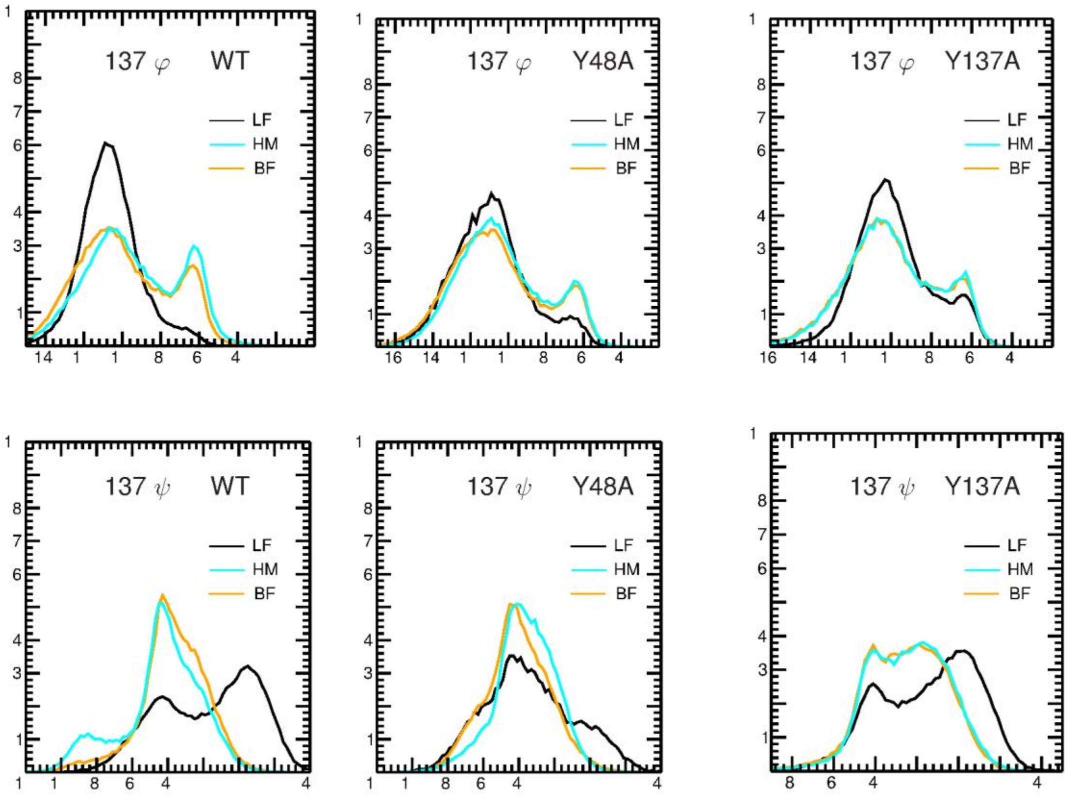
137 φ WT
137 φ Y48A
137 φ Y137A
137 ψ WT
137 ψ Y48A
137 ψ Y137A
LF
HM
BF

(b)

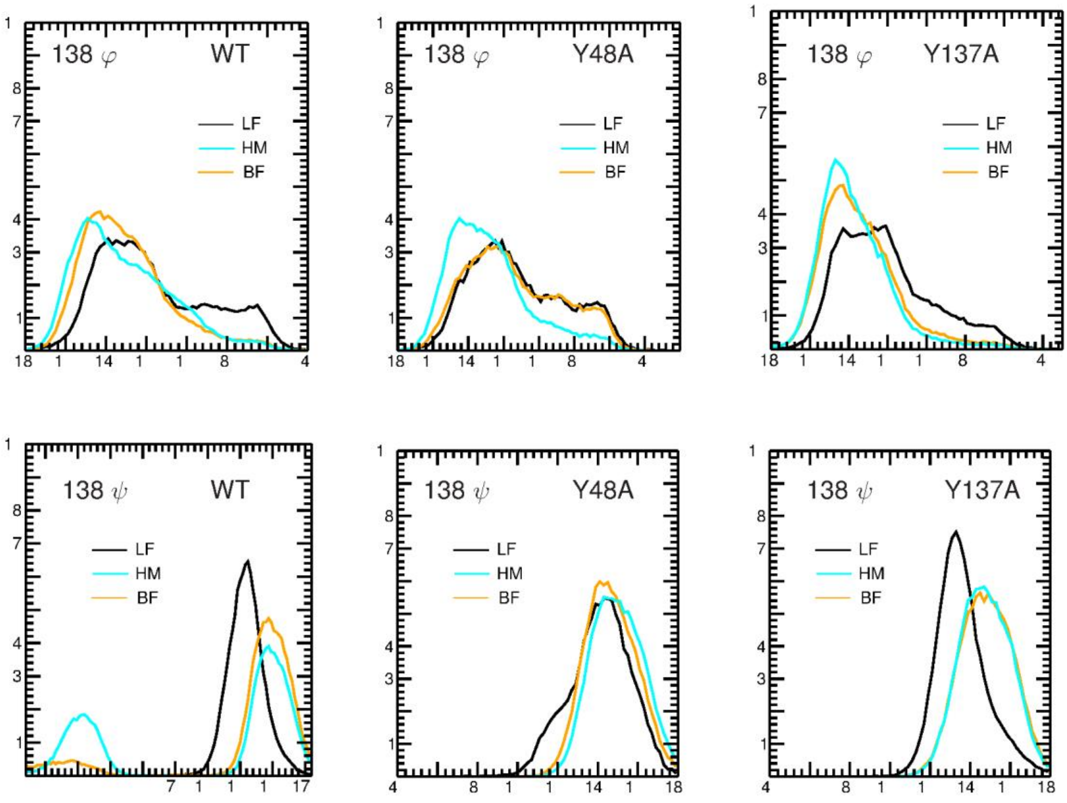

(*c*)

Figure S6 Backbone dihedral angle distributions of the oligopeptide, holding residues 136 to 138, as extracted from MD simulations of WT FimH and theY48A and Y137A mutant FimH, either in its ligand-free (LF) state or bound to either ligand BF or HM. The probability <x> of finding the backbone dihedral angles φ and ψ of the residues (*a*) 136, (*b*) 137, and (*c*) 138, at a certain value is plotted as extracted from ligand-free (black), the HM (cyan) and the BF (orange) bound simulations. The distributions are calculated over the total of 3x50 ns trajectories.

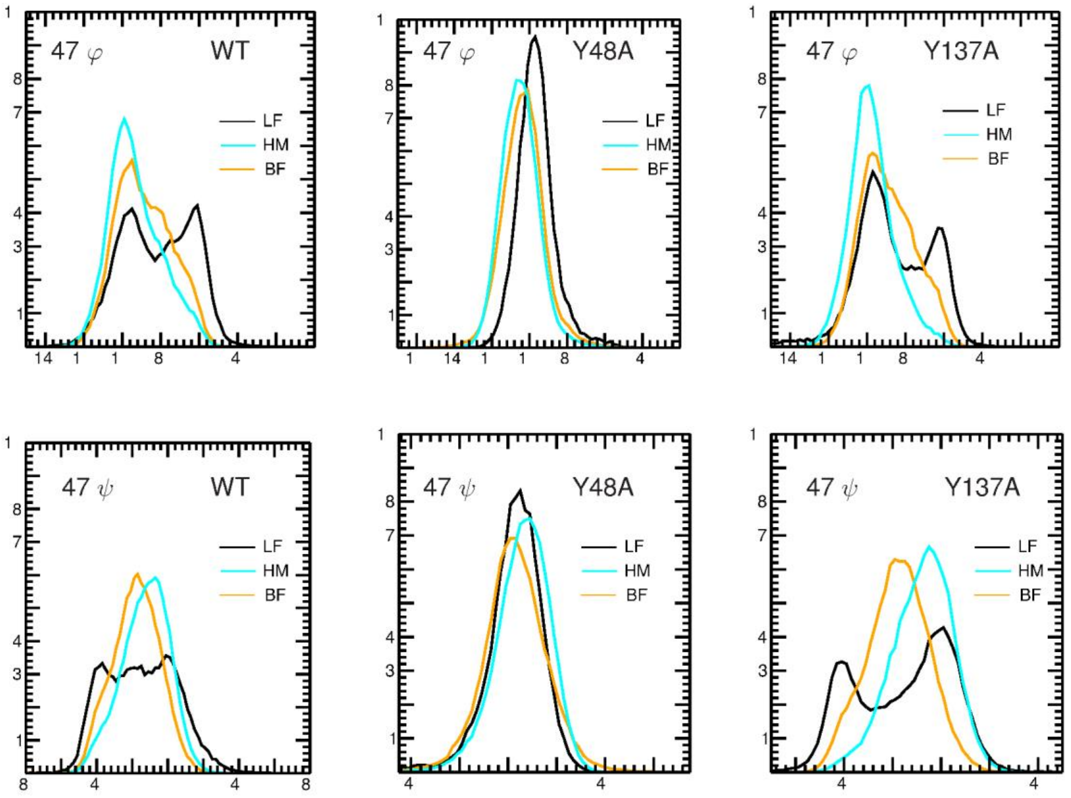
47 φ WT
47 φ Y48A
47 φ Y137A
47 ψ WT
47 ψ Y48A
47 ψ Y137A
LF
HM
BF

(a)

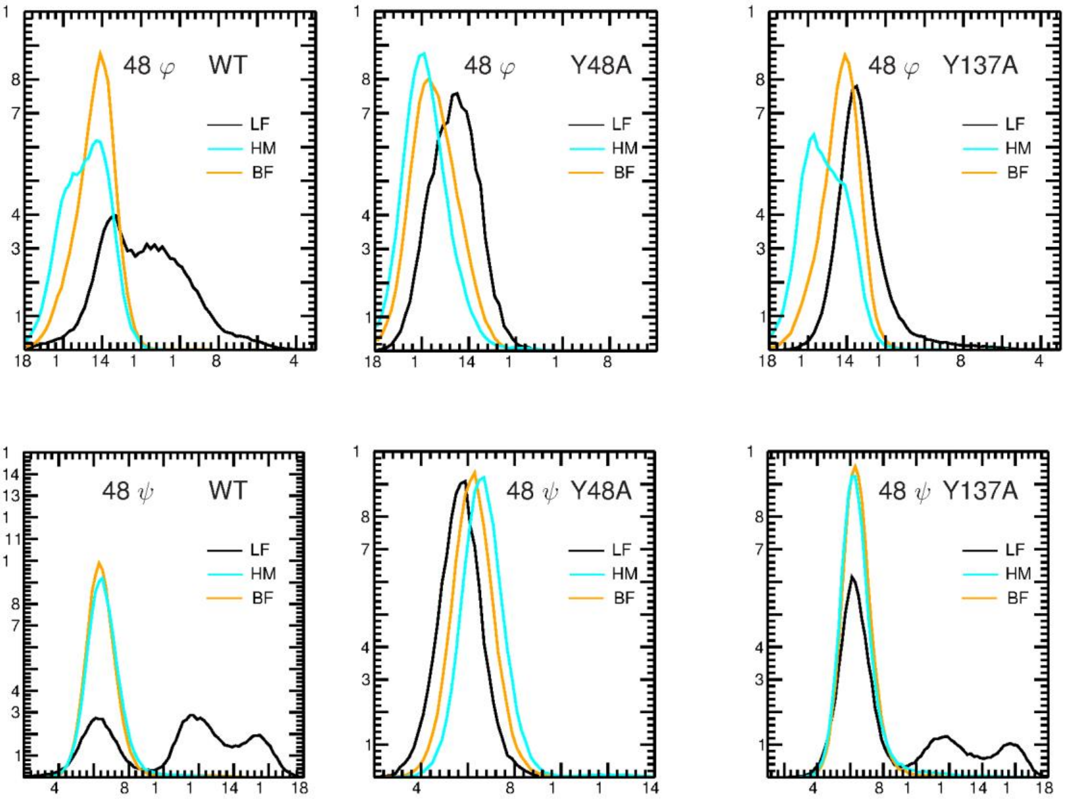
48 φ WT
48 φ Y48A
48 φ Y137A
48 ψ WT
48 ψ Y48A
48 ψ Y137A
LF
HM
BF

(b)

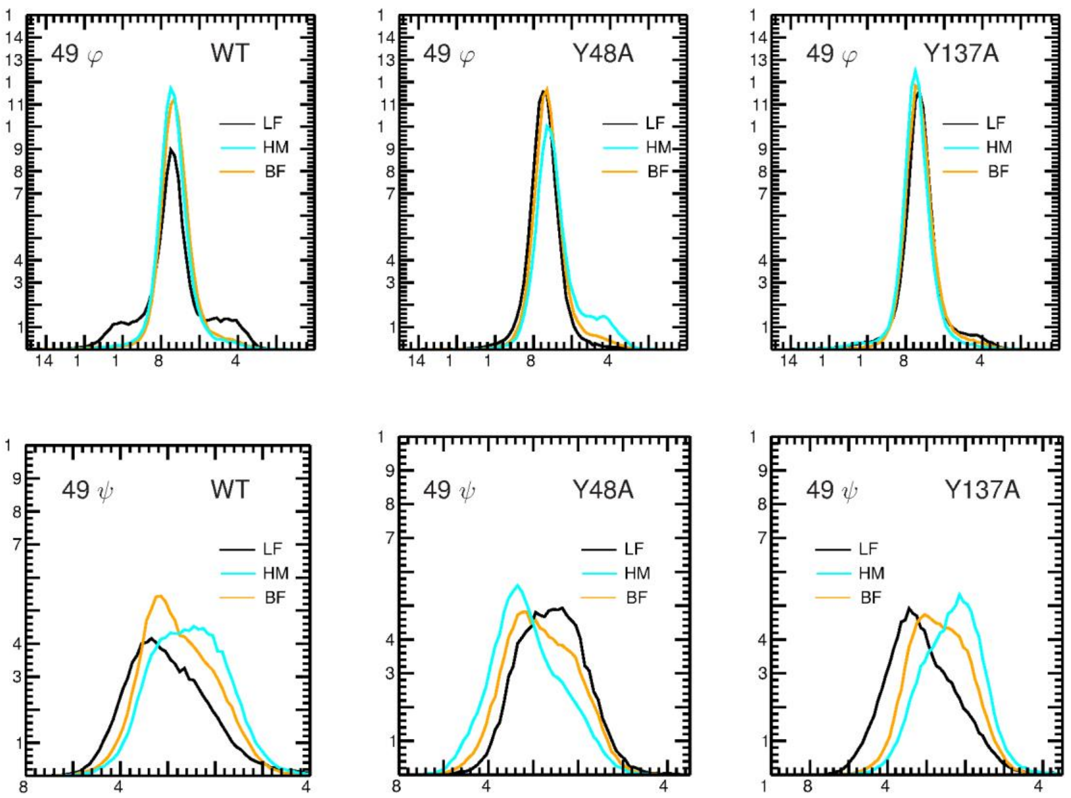

(c)

Figure S7 Backbone dihedral angle distributions of the the oligopeptide, holding residues 47 to 49, region as extracted from MD simulations of FimH WT, Tyr48Ala and Tyr137Ala mutant either in its ligand-free (LF) state or bound to either BF or HM. The probability <x> of finding the backbone dihedral angles φ and ψ of the residues (*a*) 47, (*b*) 48, and (*c*) 49 at a certain value is plotted as extracted from ligand-free (black), the HM (cyan) and the BF (orange) bound simulations. The distributions are calculated over the total of 3x50 ns trajectories.
